# Variety-Specific Transcriptional and Alternative Splicing Regulations Modulate Salt Tolerance in Rice from Early Stage of Stress

**DOI:** 10.1186/s12284-022-00599-9

**Published:** 2022-11-03

**Authors:** Guihua Jian, Yujian Mo, Yan Hu, Yongxiang Huang, Lei Ren, Yueqin Zhang, Hanqiao Hu, Shuangxi Zhou, Gang Liu, Jianfu Guo, Yu Ling

**Affiliations:** 1 College of Coastal Agricultural Sciences, Guangdong Ocean University, Zhanjiang, 524088 People’s Republic of China; 2 South China Branch of National Saline-Alkali Tolerant Rice Technology Innovation Center, Zhanjiang, 524088 People’s Republic of China; 3 Department of Biological Sciences, Macquarie University, North Ryde, NSW 2019 Australia; 4 Hubei Key Laboratory of Food Crop Germplasm and Genetic Improvement, Food Crops Institute, Hubei Academy of Agricultural Sciences, Wuhan, 430064 People’s Republic of China

**Keywords:** Rice, Salt stress, mRNA alternative splicing, Gene expression, Chloroplast, ROS

## Abstract

Salt stress poses physiological drought, ionic toxicity and oxidative stress to plants, which causes premature senescence and death of the leaves if the stress sustained. Salt tolerance varied between different rice varieties, but how different rice varieties respond at the early stage of salt stress has been seldom studied comprehensively. By employing third generation sequencing technology, we compared gene expressional changes in leaves of three rice varieties that varied in their level of tolerance after salt stress treatment for 6 h. Commonly up-regulated genes in all rice varieties were related to water shortage response and carbon and amino acids metabolism at the early stage of salt stress, while reactive oxygen species cleavage genes were induced more in salt-tolerant rice. Unexpectedly, genes involved in chloroplast development and photosynthesis were more significantly down-regulated in the two salt tolerant rice varieties ‘C34’ and ‘Nona Bokra’. At the same time, genes coding ribosomal protein were suppressed to a more severe extent in the salt-sensitive rice variety ‘IR29’. Interestingly, not only variety-specific gene transcriptional regulation, but also variety-specific mRNA alternative splicing, on both coding and long-noncoding genes, were found at the early stage of salt stress. In summary, differential regulation in gene expression at both transcriptional and post-transcriptional levels, determine and fine-tune the observed response in level of damage in leaves of specific rice genotypes at early stage of salt stress.

**Supplementary Information:**

The online version contains supplementary material available at 10.1186/s12284-022-00599-9.

## Introduction

Rice (*Oryza sativa* L.) is one of most important crops on the Earth. There are more than 150 million hectares of land here for planting rice, a large proportion of which is salinized to some degree or are at risk of salinization (Solis et al. 2020). Rice is a salt sensitive crop, and producing of salt tolerant rice is important for food security. Uncovering the mechanism underlying variations of salt tolerance between different rice varieties is important for breeding of elite salt tolerant rice genotypes.

Salt over-accumulation in soils will initially causes osmotic stress in plant cells, and then ionic imbalance and Na^+^ toxicity when the plant absorbed saline water from soils through its roots (Formentin et al. 2018; Munns and Tester 2008). Disruption of photosynthesis and accumulation of reactive oxygen species (ROS) are physiological and physiochemical changes usually found after salt stress (Formentin et al. 2018). In addition, the carbon and nitrogen metabolic processes were reorganized in plants under moderate salt stress, but disturbed under severe salt stress (Munns and Tester 2008). Therefore, some plants generated salt tolerant capacities during their evolution for adaptation to live in salinized soils. For example, generation of osmotic regulators would sustain water-uptake capacity of roots from the low water potential soil conditions in some mangrove species (Jiang et al. 2022).On the other hands, sequence variations and/or expressional changes of ion transporters that endure relative low accumulation of Na^+^ in the cytoplasm of cells could lighten the Na^+^ toxicity and finally increase the salt tolerance of plants (Kobayashi et al. 2017; Obata et al. 2007; Oomen et al. 2012; Ren et al. 2005; Shen et al. 2015). In addition, upregulation of enzymes and non-enzymatic antioxidants responding for suppressing ROS over-accumulation directly and indirectly would also enhance salt tolerance in rice (Kaur et al. 2016; Steffens 2014; Wang et al. 2018b; Zhou et al. 2018). In rice, Many transcription factors, including *OsCOIN* (Liu et al. 2007), *OsbZIP71* and *OsbZIP23* (Sun et al. 2010), *OsMYB2* (Yang et al. 2012), *DST* and *DCA1* (Cui et al. 2015), have been found to be regulate salt tolerance by controlling expressions of functional genes discussed above.

Transcriptomic changes after salt stress in rice have been studied extremely in recent years. Thus, most of which were focused on that happened several days or weeks after salt stress. Transcriptomes between different rice varieties were compared at 1 day (Mirdar Mansuri et al. 2019), 2 and 3 days (Prusty et al. 2018; Razzaque et al. 2017; Wang et al. 2018a), 7 days (Cartagena et al. 2021; Walia et al. 2005), 8 days (Li et al. 2017; Zhang et al. 2022), and totally 14 days (Wang et al. 2016b) after salt stress. Few overlapped DEGs between different rice varieties were found in most of these studies. For example, salt up-regulated genes involved in several pathways underlying salt tolerance, including ABA-mediated cellular lipid and fatty acid metabolic processes and cytoplasmic transport, sequestration by vacuoles, detoxification and cell-wall remodeling in shoots, and oxidation–reduction reactions in roots of the tolerant rice variety (Wang et al. 2016b). Furthermore, DEGs from ‘Horkuch’ background (salt tolerant) and that of ‘IR29’ background (salt sensitive) were enriched in different GO terms. Interestingly, they found down-regulated genes were enriched in photosynthesis only in the ‘IR29’ background populations (Razzaque et al. 2017). Transcription regulators including *NAC*, *WRKY* and *MYB* family were tended to be down-regulated in ‘IR29’, while more transporters were regulated at gene transcriptional level in the salt tolerant varieties ‘Mulai’ (Cartagena et al. 2021). However, transcriptomes at relatively late stages of salt stress may be the result of earlier adjustments in gene expression levels by salt stress (or even caused by cell damage), and therefore inadequate in detecting changes leading to the defensive outcome in survivability.

Long non-coding RNA (lncRNA) is the mRNA more than 200 nucleotides in length that are not translated into protein (Liu et al. 2012; Rinn and Chang 2012). These have been suggested to be ‘transcriptional noise’ without biological functions in earlier studies (Huttenhofer et al. 2005). This view changed later since lots of lncRNAs are expressed in plants playing important roles, for example, the expression level of a lncRNA gene was changed by SNP resulting in photoperiod-sensitive male sterility (Ding et al. 2012). A considerable number of conserved lncRNAs in rice and maize which may be functionally related to agricultural traits (Wang et al. 2015). Moreover, lncRNAs were considered to be a potential memory factor of drought stress in rice (Li et al. 2019). LncRNAs can affect target proteins in *cis-* or *trans*-manners (Faghihi and Wahlestedt 2009; Ulitsky and Bartel 2013) at multiple levels, from chromatin structure modulation to transcriptional, post-transcriptional, translational and post-translational regulations (Fatica and Bozzoni 2014; Fonouni-Farde et al. 2021).

Accumulating studies indicated that alternative splicing (AS) is a key regulatory mechanism in eukaryotes generating varied transcripts to make the organism adapt to internal and external cues. Alternatively spliced transcripts could be either translatable or untranslatable. The untranslatable isoform could function by regulating the expression of the translatable transcript. Therefore, the abundance of protein and its variant (s) could be changed through AS regulation of their coding genes. Such regulation of functional proteins will finally contribute to different growth and responses to salt stress between rice varieties (de Francisco Amorim et al. 2018; Eckardt 2013; Kornblihtt 2014). Most recent studies considered that AS play essential roles in plant response to temperature disturbance (Dikaya et al. 2021; Lin and Zhu 2021; Ling et al. 2021). Alternative splicing (AS) of pre-mRNAs has been reported to be involved in plant growth and environmental cues, including biotic and abiotic stresses (Ling et al. 2017, 2018; Palusa et al. 2007). Different genes in the same metabolism pathways could undergo differential intensity of AS regulation, which may amplify or compromise the intensity of original stimuli (Ling et al. 2018; Liu et al. 2013; Reddy et al. 2013). Shankar et al (2016) analyzed integrated-transcriptome by using mixed RNA samples from three time points (3 h, 6 h and 12 h, respectively) after 200 mM NaCl treatment and found that there were a considerable number of AS events in samples with- and without salt treatment. The number of AS events was reduced after salt stress, suggesting that mRNA splicing processes were regulated by salt stress. Supportively, it has been demonstrated most recently that mutation of a SR protein, RS33, in rice disturbed pre-mRNA splicing of some genes and affect cold and salt tolerances of rice (Butt et al. 2022).

Here, we have used three rice genotypes which possess different levels of salt stress response for comprehensive studying the gene transcription and pre-mRNA splicing regulation in response to salt stress. The rice seedlings were treated with 100 mM NaCl (this treatment will not kill any rice seedlings in used until several days (Hu et al. 2020)) for 6 h (a point that were expected to be able to detect lots of AS events after abiotic stress initiation (AlShareef et al. 2017; Butt et al. 2022; Ling et al. 2017)) and then collected for analysis. Extensive data mining, confirmation, and bioinformatics analysis was carried out after the third generation long-read RNA-sequencing (RNA-Seq) to uncover the characteristics of gene expressional regulation in these rice varieties. Common genes regulated in the genotypes and variety-specific ones through transcription and pre-mRNA AS and their potential physiological functions were discussed.

## Materials and Methods

### Plant Materials, Growth Conditions, Stress Treatment

Plant materials, growth conditions and treatments were modified a little from our previous study (Hu et al. 2020). Two popular rice genotypes, ‘NB’ (salt tolerant) and ‘IR29″ (salt sensitive), together with a novel salt tolerant rice sample that possesses different salt tolerant mechanism from that of ‘NB’ were used in this study. Seeds were surface sterilized by incubation in 75% ethanol for 2 min, then washed thoroughly with distilled water. The sterilized seeds were germinated on moist tissue paper. The germinated seedlings were transferred for further growth with 1/4 strength Hoagland solution. This hydroponic experiment was carried out in growth room with 26 °C ~ 28 °C and 16 h photoperiod. For RNA-Seq and PCR experiments, seedlings were treated with 100 mM NaCl for 6 h, and their leaves were then submerged into liquid nitrogen for 3 min and stored at -80℃ or sent for RNA-Seq directly in dry ice. For ROS contents measurement, seedlings were treated with 100 mM NaCl for 5d. Three replicates were done for RNA-Seq and ROS measurements.

### ROS Measurement

Visualization of reactive oxygen species (ROS) was detected by nitro blue tetrazolium (NBT) staining. In brief, rice leaves were first cut into sections and then immersed in50 mL staining solution (0.1% (w/v) NBT, 10 mM sodium azide, 50 mM potassium phosphate, pH 6.4), followed by infiltration through vacuum (∼100–150 mbar) for 10 min. The incubation was conducted in a growth chamber in the dark overnight. H_2_O_2_ contents were measured in the second leaf of each rice plant after salt stress for 0 h, 6 h and 5d, respectively. The H_2_O_2_ contents method were modified a little from (Zafar et al. 2020).

### Total RNA Extraction and Library Preparation

The total RNA was extracted by using RNA Extraction Kit (Tiangen, Beijing, China). The quality of RNA was detected by RNase-free agarose gel electrophoresis. The concentration and purity of the total RNA samples were detected by NanoDrop 2000, followed by calculation of 28S/18S ratio and RIN value through Agilent 2100 (Agilent Technologies, Santa Clara, Technologies, CA, United States). Poly (A) mRNA was purified and enriched from total RNA by mRNA capture beads (kits bought from Vazyme, Nanjing, China). Reverse transcription primers were used to bind to the poly-A tail of mRNA by annealing. Double cDNA strand was amplified by PCR amplification primers. The DNA damage repair and plus-A reaction were done by using NEBNext FFPE DNA Repair Mix and NEBNext Ultra II End Repair/dA-Tailing Module, respectively. Connections of sequencing adaptors were done by using the SQK-LSK109 kit (ONT). A total of 18 RNA-Seq libraries were constructed and sequenced.

### RNA-Seq, Reads Processing and Data Analysis

Transcriptome sequencing was done by Biomarker Technologies (Beijing, China) using PromethlON 48 platform (Oxford Nanopore Technologies, UK) including the processing of Reads. In brief, raw reads were firstly filtered with minimum average read Q score = 7. And then ribosomal RNAs were removed through rRNA database mapping. Full-length and non-chimeric (FLNC) transcripts were determined by searching for primer at both ends of reads. Clusters of FLNC transcripts were obtained after mapping to rice reference genome with mimimap2. After that, mapped reads were further collapsed by cDNA_Cupcake package (https://github.com/Magdoll/cDNA_Cupcake) with min-coverage = 85% and min-identity = 90%.

The basic RNA-Seq analysis has been run through the platform Biomarker Cloud. DESeq was used for differential expression analysis between samples and treatments. Transcripts in a comparison with Fold_Change ≥ 2 and FDR (false discovery rate) < 0.01 were considered to be significantly differential expressional events. Specifically, Fold _Change represents the ratio of expression between two samples (groups) and FDR is measured according to (Klipper-Aurbach et al. 1995). Further comparisons and visualizations of the sequencing data were done through the analysis platform of Biomarker Cloud and program R.

### LncRNA and Candidate Targets Prediction

LncRNAs were predicted from four different platforms, (Pfam, CNCI, PCP and CPAT). A transcript longer than 199 nt and predicted to be non-coding sequence was considered to be a lncRNA candidate. The final lncRNAs were those which overlapped in all prediction platforms. Similarly, candidate targets of lncRNAs were predicted, through two different methods. Genes located on a chromosome within ± 100 kb around a lncRNA could be considered as its *cis*-targets. *Trans*-target was screened by the free energy of sequence complementarity between a lncRNA and a coding gene. A gene with free energy of pairing sites less than -0.1 (the standardized free energy threshold) could be considered as a target.

### qRT-PCR and RT-PCR Validations

Reverse-transcription PCR (RT-PCR) and reverse-transcription quantitative PCR (RT-qPCR) were performed as previously described (Ling et al. 2017). For reverse-transcription quantitative PCR (RT-qPCR), DNA digestion of total RNA samples was performed after RNA extraction using an RNase-Free DNase Set (Vazyme,catalogno.R223-01) following the manufacturer’s protocol. The total RNA was reverse transcribed using a SuperScript First-Strand Synthesis System for RT-qPCR (Invitrogen) to generate cDNA. The qPCR was performed using Power SYBR Green PCR Master Mix (Yeasen Biotech, catalog no. 11201ES08) under the following conditions: 95 °C for 10 min, then cycles of 95 °C for 15 s, 60 °C for 1 min. Statistical analysis of gene expression in qRT-PCR was performed by Microsoft Excel2013. In RT-PCR validation of AS events, primers flanking different exons were used.

## Results

### Phenotypes and ROS Contents in Leaves of Different Rice Varieties After Salt Stress

**Fig. 1:**
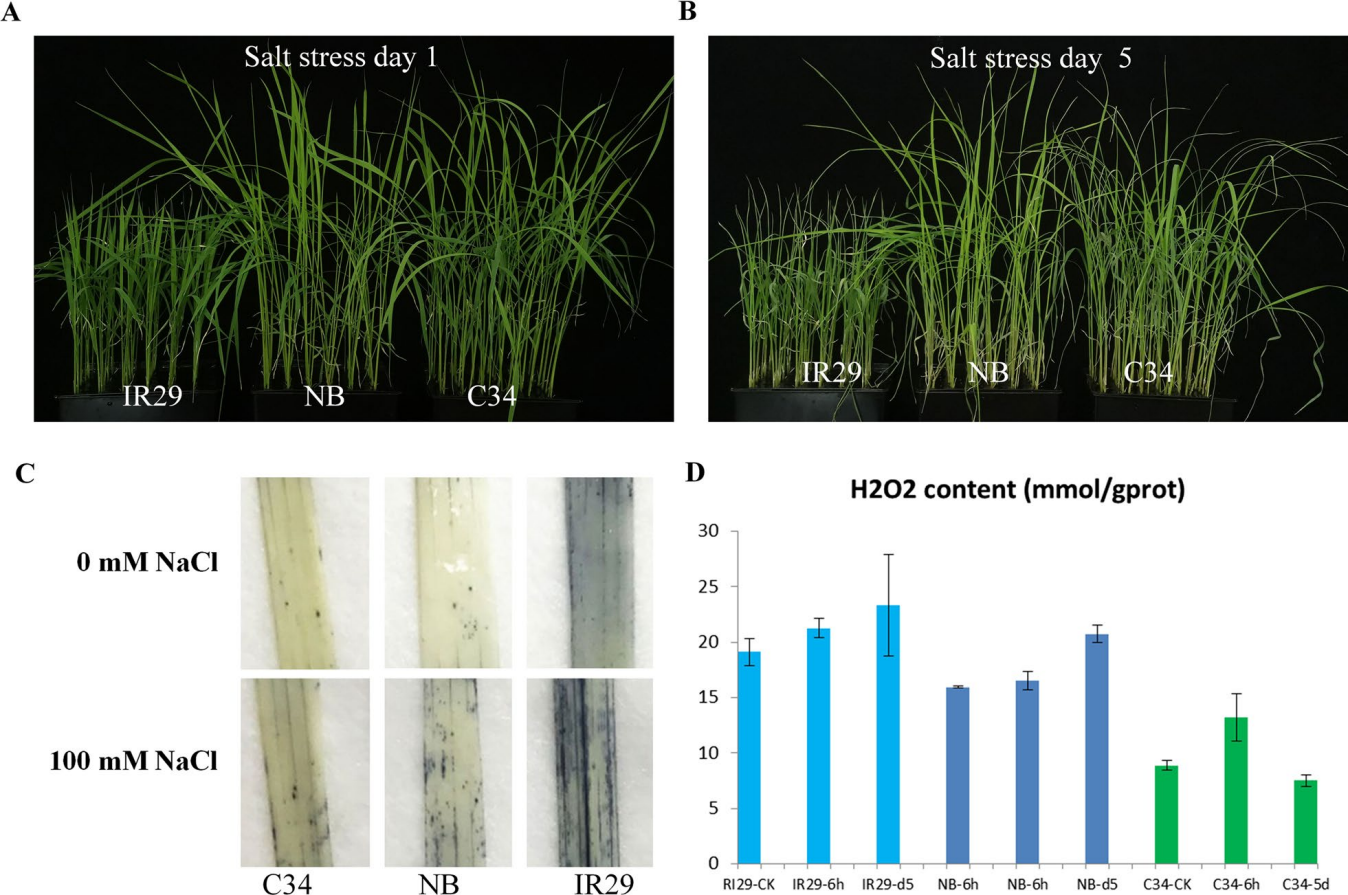
Phenotype and ROS accumulation of rice leaves after salt stress. **A**. Phenotype of the three rice varieties after 100 mM NaCl treated for 1 day. **B**. Phenotype of the three rice varieties after 100 mM NaCl treated for 5 days. **C**. ROS staining on leaves without NaCl treatment. Lower panels, ROS staining on leaves from seedlings treated with 100 mM NaCl for 5 days. Sample IDs were indicated. **D**. H_2_O_2_ contents in the second leaf of each rice varieties. Salt treatment durations were 0 h, 6 h and 5d, respectively

In an earlier study, we have screened several salt-tolerant rice varieties, including the two rice landraces collected from shores of South China. It has been demonstrated that Chlorophyll contents and survival rates decreased at different rates after salt stress treatments for 10 days (Hu et al. 2020). We are curious to know how these rice varieties performed at early stage of salt stress. We observed phenotypes of some of these rice varieties after salt stress for 1 day and 5 days. Our result demonstrated that no significant changes were found but only growth rates reduced after salt stress for 1 day (Fig. 1A). After 5 days, salt stress treated rice plants show more severe wilt symptoms on leaf apexes, especially in that of ‘IR29’, which contained more ROS (Fig. 1B, C).

### High Quality of Long-Read RNA-Seq Data

To discover the transcription and pre-mRNA splicing variation of leaves from different rice varieties at early stage of salt stress, we harvested the salt stressed rice plants after application of salt stress for 6 h. Then, we employed the ONT long-read sequencing platform to explore the sequence information in our study. Details about biological replicates and quality control are provided in the supplementary (Additional file 1: Fig. S1, Additional file 2: Fig. S2 and Additional file 3: Fig. S3). The transcriptome sequencing of 18 rice samples generated about 22 ~ 46 million clean reads with a base calling accuracy > 97.40% for each library. The mean-length of transcripts varied between 736 and 926 bp. The full-length read percentages were about 77.8% ~ 85.24% (Table 1, Additional file 1: Fig. S4). These results suggested we have got relative high quality of RNA-Seq data. Long-read sequencing technologies remove the disadvantage of short-read assembly process which is necessary in older sequencing technologies (Cui et al. 2020). So the high quality of long-read RNA-Seq data and technical advantages enabled us to do gene expressional analysis more accurately, especially in the aspect of pre-mRNA splicing analysis.

**Table 1:** An overview of RNA-Seq data quality

Sample	Total Read	Mapped read	Mapped rate	Fulllength_Read number	Fulllength_Read ratio (%)	N50	MeanLength
C34_CK1	2,530,965	2,488,745	0.983319	2,138,003	84.47	957	899
C34_CK2	2,983,480	2,932,989	0.983076	2,474,787	82.95	959	898
C34_CK3	4,482,464	4,400,296	0.981669	3,574,214	79.74	920	863
C34_T1	2,693,869	2,629,333	0.976043	2,244,497	83.32	928	873
C34_T2	4,603,445	4,489,252	0.975194	3,862,941	83.91	862	817
C34_T3	2,436,353	2,380,347	0.977012	2,046,120	83.98	892	841
IR29_CK1	2,195,403	2,165,350	0.986311	1,858,616	84.66	946	891
IR29_CK2	4,680,978	4,587,375	0.980004	3,957,768	84.55	836	790
IR29_CK3	2,368,677	2,332,909	0.9849	2,010,540	84.88	944	892
IR29_T1	3,624,356	3,508,433	0.968016	3,066,407	84.61	760	739
IR29_T2	4,590,649	4,493,456	0.978828	3,849,527	83.86	874	826
IR29_T3	4,115,444	4,008,356	0.973979	3,460,835	84.09	824	784
Nona_Bokra_CK1	2,937,994	2,888,265	0.983074	2,504,408	85.24	937	883
Nona_Bokra_CK2	2,682,777	2,641,913	0.984768	2,234,593	83.29	983	919
Nona_Bokra_CK3	2,513,349	2,471,724	0.983438	2,107,576	83.86	889	840
Nona_Bokra_T1	2,235,032	2,192,326	0.980892	1,893,180	84.70	928	876
Nona_Bokra_T2	4,131,432	4,069,107	0.984914	3,292,952	79.70	984	926
Nona_Bokra_T3	4,153,922	4,050,561	0.975117	3,500,880	84.28	768	736

### Different Expressional Regulations in Different Rice Varieties

**Fig. 2:**
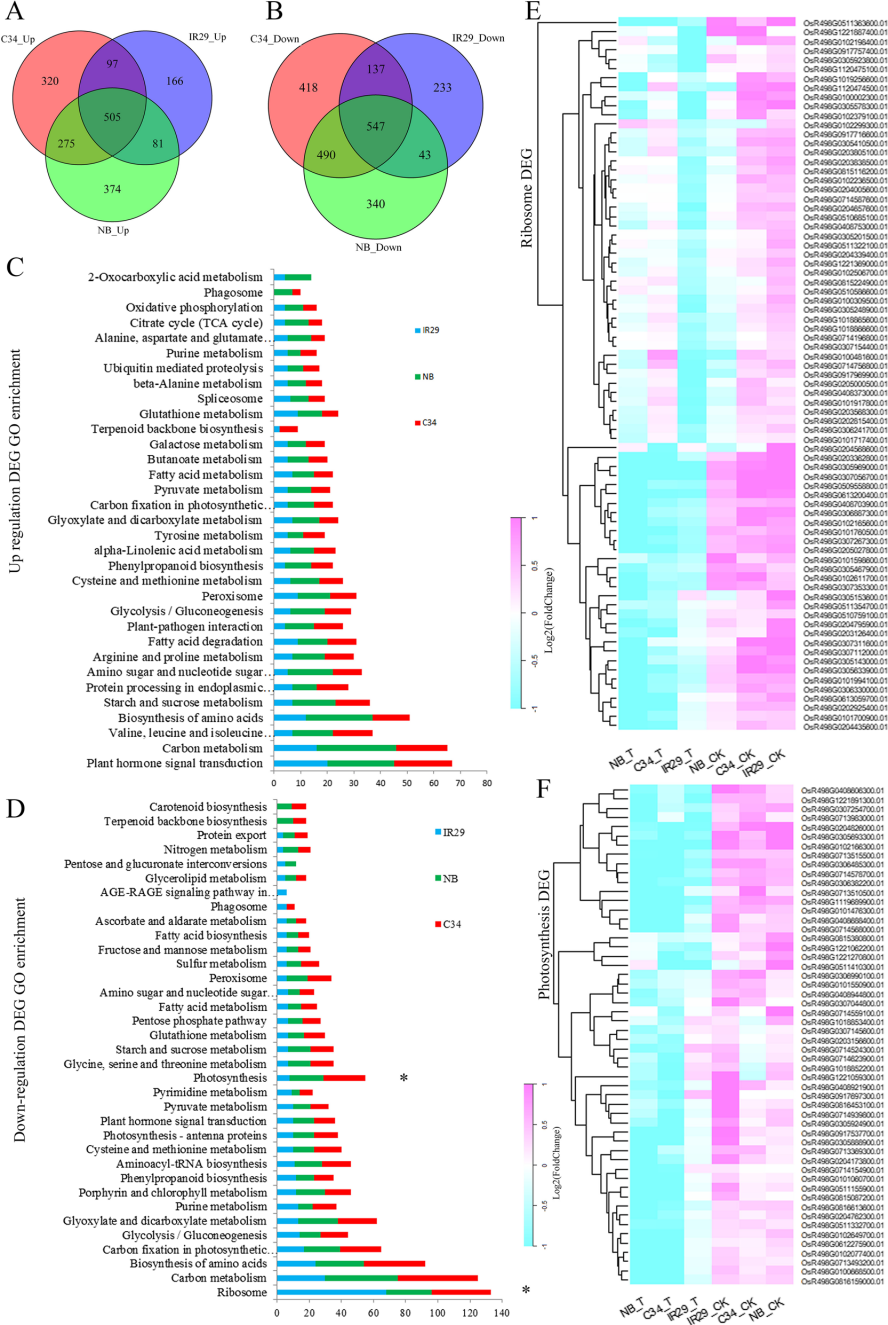
Numbers and GO enrichments of DEGs in different rice varieties. **A**. Numbers of common and variety-specific up-regulated genes. **B**. Numbers of common and variety-specific down-regulated genes. **C**. Molecular function enrichment of up-regulated genes in different rice varieties (Only top30 enriched terms were shown). **D**. Molecular function enrichment of down-regulated genes in different rice varieties (Only top30 enriched terms were shown). Stars ‘*’ mark enriched GO terms ‘Photosynthesis’ and ‘Ribosome’, respectively. **E**. DEGs enriched in GO term ‘Ribosome’. The color key stands for change fold of gene expression shown in log2 style. **F**. DEGs enriched in GO term ‘Photosynthesis’. The color key stands for change fold of gene expression shown in log2 style

A total of 15,865 and 16,334 genes were expressed in the three rice varieties under control conditions and salt stress conditions, respectively. The South China landrace ‘C34’ possessed most of variety-specific expressed genes under both control and salt-treatment conditions (Additional file 5: Fig. S5). DEG analysis of the RNA-Seq data demonstrated that there were totally more than 2200 down-regulated and 1800 up-regulated genes by salt stress (Fig. 2A, B). The number of DEG in ‘C34’ and ‘NB’ was more than that of the salt-sensitive sample ‘IR29’, suggesting more activated modulation at transcriptional level in these salt-tolerant rice varieties when sensing the salt stress signal. After gene ontology (GO) analysis, we measured the number of regulated genes in each functional term and integrated the top 30 enriched terms from each rice variety (Additional file 9: Table). Genes encoding proteins with molecular function in carbon metabolism and amino acid biosynthetic activities were enriched in both up- and down-regulated gene clusters by salt stress (Fig. 1C, D), indicating acute adjustments of carbohydrate and protein biosynthesis and degradation were carried out in all rice varieties after salt stress for 6 h. Generally, the up-regulated genes in ‘IR29’ were less than that of the two salt-tolerant rice varieties in many enriched terms.

Up-regulated genes were also highly enriched in molecular function terms of “Plant hormone signal transduction”, “Starch and sucrose metabolism”, “Amino sugar and nucleotide sugar metabolism”, “Valine, leucine and isoleucine degradation” and “Peroxisome” (Fig. 1C). Furthermore, “Terpenoid backbone biosynthesis” being enriched the top 30 GO enrichment of up-regulated genes in ‘C34’, but not or only slightly accumulated in either ‘IR29’ or ‘NB’, whereas “2-Oxocarboxylic acid metabolism” was only found in top 30 enriched up-regulated genes of ‘NB’ and ‘IR29’, but not in that of ‘C34’. On the other hand, “Pentose and glucuronate interconversions” was the top 30 enriched terms of down-regulated genes in ‘NB’ and ‘IR29’, but not in that of ‘C34’.

In the case of down-regulated genes, highly enriched functional terms were involved in ribosome, carbon fixation in photosynthetic organisms, glyoxylate and dicarboxylate metabolism, photosynthesis and porphyrin and chlorophyll metabolism (Fig. 2D). ‘Terpenoid backbone biosynthesis’ and ‘Carotenoid biosynthesis’ were two of top 30 terms of down-regulation genes detected only in the two salt tolerant rice varieties, while other genes involved in GO term ‘‘Terpenoid backbone biosynthesis’’ were only in top 30 enrichment of ‘C34’ and ‘IR29’. It is noteworthy that many more genes with functional term of “Ribosome” were suppressed in ‘IR29’, while the numbers of down-regulated genes with other GO terms were generally less than that of ‘C34’ and ‘NB’ (Fig. 2D, E). More interestingly, many more genes involved in photosynthesis were down-regulated in ‘C34’ and ‘NB’ when compared with ‘IR29’ (Fig. 2D, F), even though the chlorosis symptom and pre-mature senescence were more obvious in ‘IR29’ if sustained salt stress was applied (Hu et al. 2020).

### Common and Specific Biological Processes Affected by Salt Stress in Different Rice Varieties

**Fig. 3:**
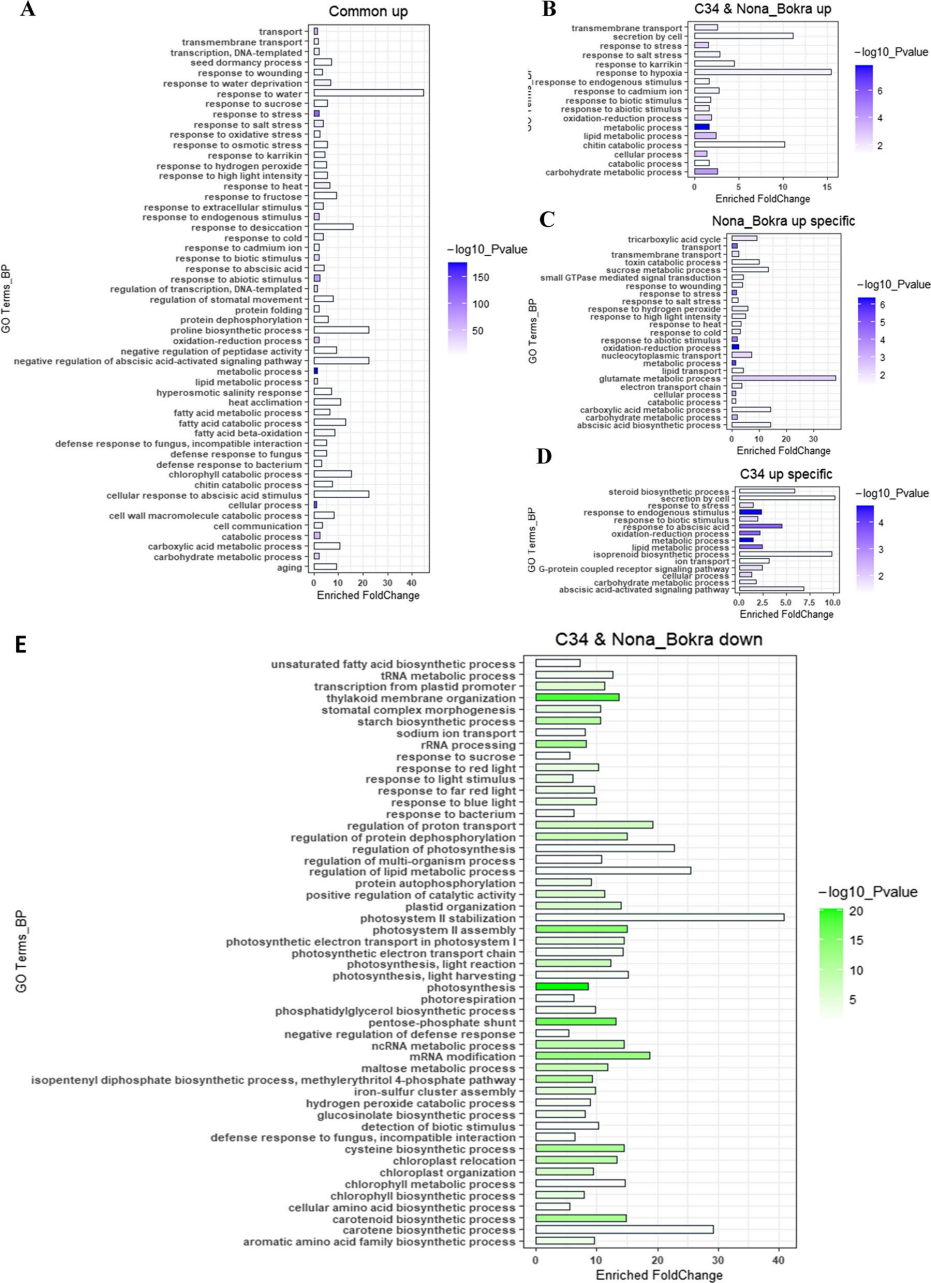
Common- and variety-specific biological process enrichments of salt stress regulated DEGs. **A**. Biological process enrichment of commonly up-regulated genes in all three rice varieties. **B**. Biological process enrichment of commonly up-regulated genes in ‘C34’ and ‘NB’. **C**. Biological process enrichment of ‘NB’ specific up-regulated genes. **D**. Biological process enrichment of ‘C34’ specific up-regulated genes. **E.** Biological process enrichment of down-regulated genes in salt-tolerant rice varieties, ‘C34’ and ‘NB’

Next, we wanted to know what biological processes these regulated genes enriched in. As shown in Fig. 3, many up-regulated genes in all three varieties were enriched in biological processes of “response to water” and “proline biosynthetic process”, together with abscisic acid (ABA) stimulus and signaling, suggesting all rice varieties tested sensed osmotic stress after salt treatment for 6 h, and ABA signaling-mediated resistance activities were triggered in these plants (Fig. 3A). Genes involved in biological processes of “response to hypoxia” and also “secretion by cell”, “chitin catabolic process” were found to be highly stimulated in ‘C34’ and ‘NB’, but not in ‘IR29’, suggesting these genes are specific up-regulated in salt-tolerant rice varieties (Fig. 3B). Specific up-regulated genes in ‘NB’ were highly enriched in glutamate metabolic process, carboxylic acid metabolic process, sucrose metabolic process, together with ABA biosynthetic process (Fig. 3C). Whereas many variety-specific up-regulated genes in ‘C34’ were involved in biological processes of “isoprenoid biosynthetic process”, “secretion by cell”, “ABA-activated signaling pathway” and “steroid biosynthetic process” (Fig. 3D). Some genes specifically induced by salt stress in ‘IR29’ were transcription regulators and stress response (Additional file 6: Fig. 6, Additional file 7: Fig. 7).

In the case of down-regulated genes, we also detected species-specific disturbance of genes. Many genes, including those involved in biological processes of “photosystem II stabilization” and “carotene biosynthetic process” and “regulation of lipid metabolic process”, “regulation of photosynthesis” and “regulation of proton transport” were down-regulated together in ‘C34’ and ‘NB’, but not in ‘IR29’ after salt stress (Fig. 3E), indicating that salt stress response in different rice varieties changed to a certain extent in these rice varieties, properly shutting down photosynthetic process at early stage of salt stress would benefit the protection of leaves from Na^+^ toxicity. Specific accumulations of different down-regulated genes were also found in each rice variety (Additional file 6: Fig. 6, Additional file 7: Fig. 7).

### Some genes Involved in Stress Response and Hormone Signaling were Regulated to a More Extent in ‘C34’ than ‘NB’

**Fig. 4:**
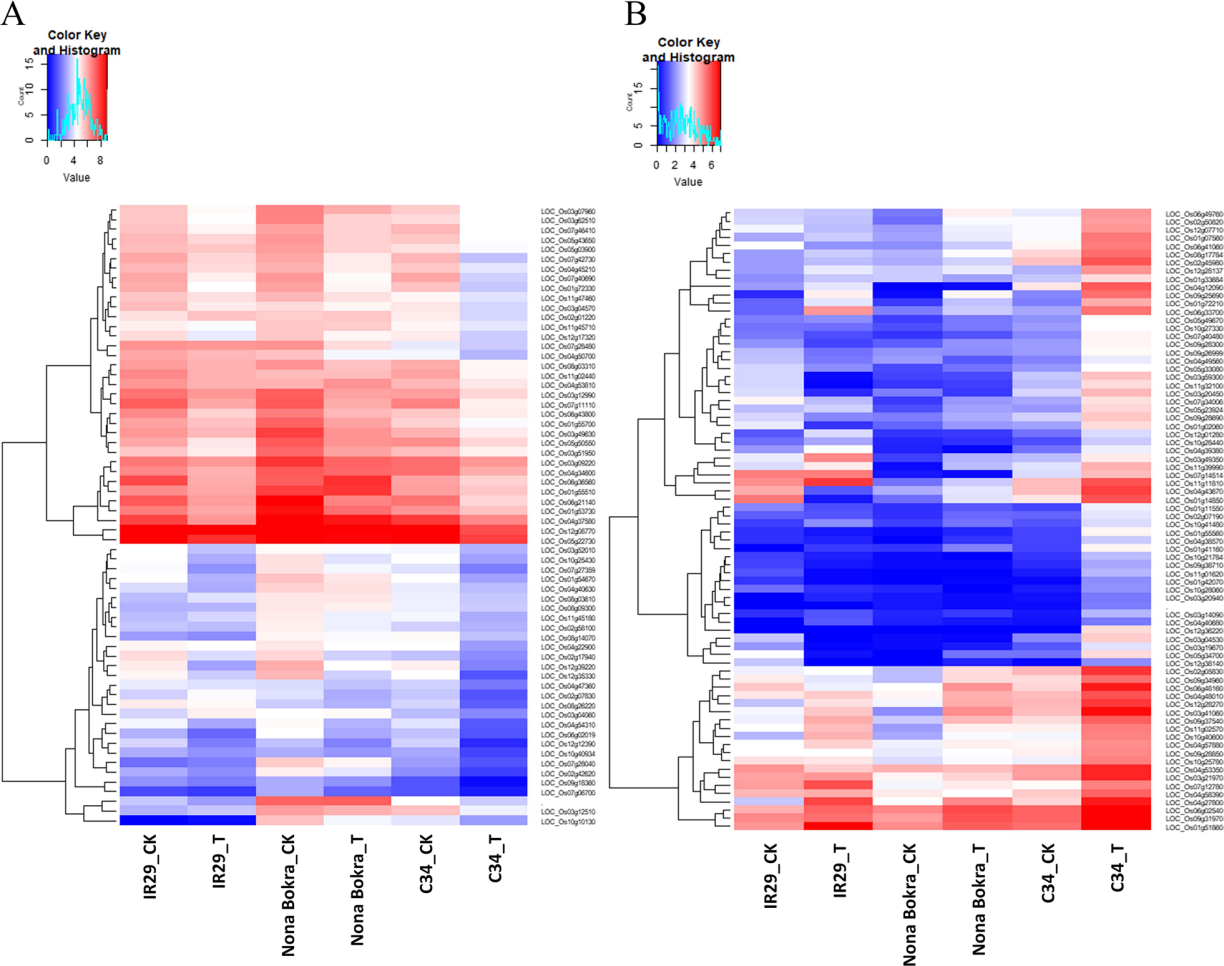
DEGs regulated to more extents in ‘C34’ than that of ‘NB’. **A**. DEGs suppressed more in ‘C34’ than in ‘NB’. **B**. DEGs up-regulated more in ‘C34’ than in ‘NB’

It has been demonstrated that in some respects, ‘C34’ performed better than ‘NB’ in tolerating salt stress (Hu et al. 2020). Here, we wanted to know what genes were regulated to greater extent in ‘C34’ in response to salt stress. In ‘C34’, 80 DEGs were specifically up-regulated or induced to a higher level in comparison with ‘NB’ after salt treatment, including several genes, which have been reported to be related to cold stress, such as *OsPP2C27* (also known as *ABIL3*, *LOC_Os01g55560*), *OsbHLH002* (*LOC_Os11g32100*) (Xia et al. 2021), *OsPCF5 (LOC_Os01g11550)* and MFS18 protein precursor (*LOC_Os01g14850*) (Yang et al. 2013), and GDSL-like genes, *LOC_Os03g19670*, *LOC_Os05g34700*, and *Os03g0683800*, which may function in amino acid transport and metabolism. The RNA-Seq data further demonstrated that few genes involved in phytohormone signaling, a brassinosteroid (BR) pathway-related gene, *LTBSG* (*LOC_Os10g25780*) (Qin et al. 2018) and two gibberellin (GA) responding genes, *GASR4* (*LOC_Os04g39110*), *GID1L2* (*LOC_Os09g28690*) were also induced more in ‘C34’ in comparison with the other two tested rice varieties. In contrast, different 67 genes were down-regulated specifically or expressed at lower levels in ‘C34’ than in ‘NB’ after salt stress treatment. This group of genes consisted of *LSD1* (*LOC_Os08g03610*), a photosystem I reaction subunit (*LOC_Os12g08770*), a pathogen defense and hypoxia-responsive gene, *WRKY62* (Fukushima et al. 2016), and so on. Interestingly, expression levels of most of these genes were also changed to a lesser extent in ‘IR29’ after salt stress (Fig. 4, Additional file 9: Table).

### Expressional Patterns of Known Salt Stress-Related Genes in Different Rice Samples

**Fig. 5:**
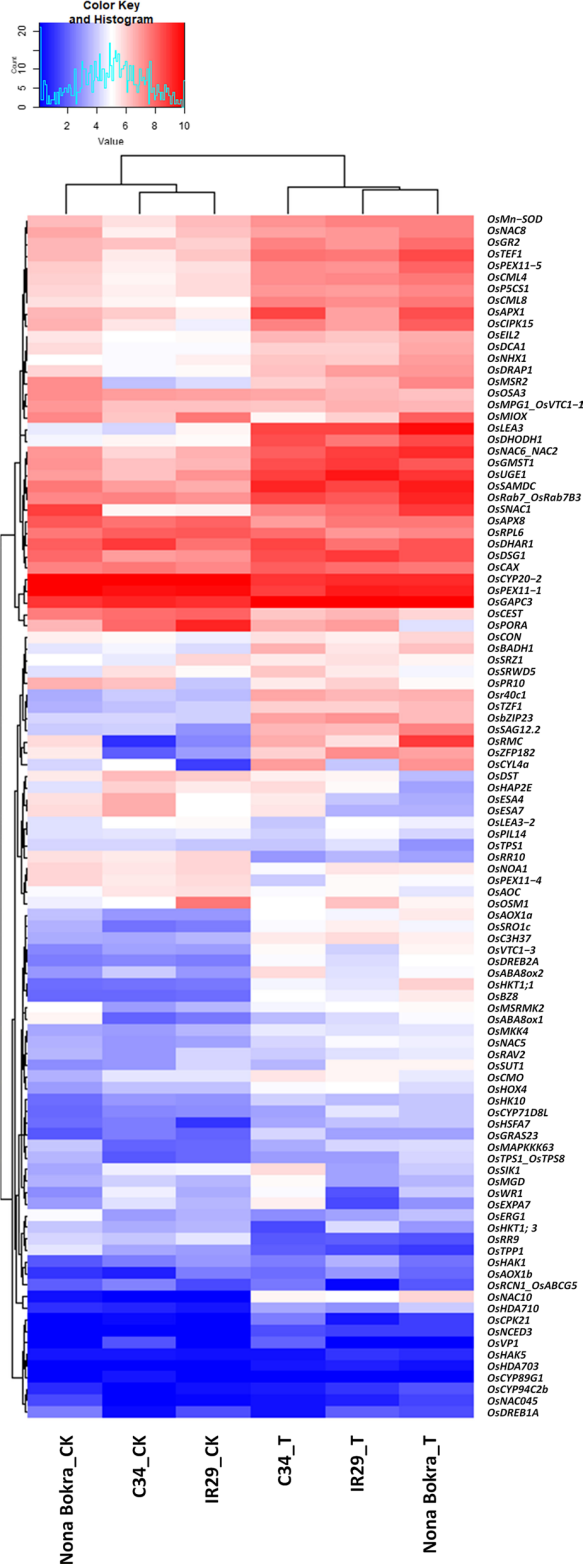
Expressional patterns of known salt tolerance-related genes in different rice varieties

Next, we summarized a list of genes that have been considered to be involved in salt resistance in rice (Akita and Cabuslay 1990; Ganie et al. 2019; Qin et al. 2020). There were more than 80 genes shown differential expression in at least one variety after salt stress (Fig. 5). Interestingly, *OsAPX1*, a gene encoding antioxidant in rice, were induced to a greater abundance in the two salt-tolerant rice samples, especially in ‘C34’. Moreover, a drought and salt related receptor-like kinase, *OsSIK1* (Ouyang et al. 2010), was accumulated to an obviously higher level in ‘C34’ both before and after salt stress, when compared to ‘NB’ and ‘IR29’. Other highly accumulated salt-related genes in ‘C34’ after salt treatment included *OsABA8ox2, OsMGD and OsEXPA,* and so on. Notably, the expression of *OsHKT1;3* (a Na^+^ transporter) and *OsDREB1A* (a stress responsive transcription factor) were suppressed in both ‘C34’ and ‘NB’but moreso in ‘C34’ than in ‘NB’. Some other salt-tolerant genes were induced more in ‘NB’ than in ‘C34’, including *OsNAC10, OsHKT1;1* and *OsRMC.*

### Expressional Variation of lncRNAs in Different Varieties

**Fig. 6:**
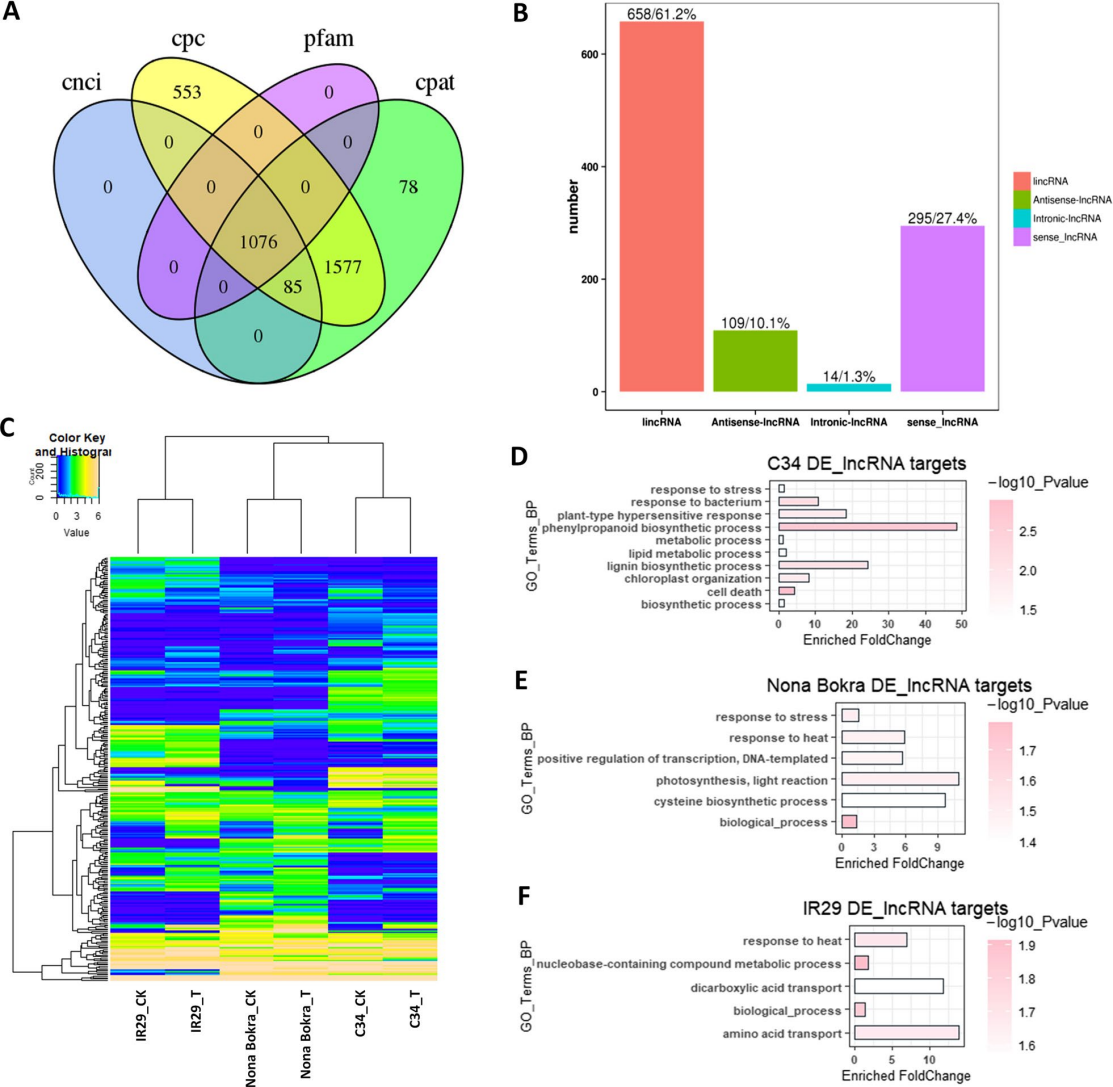
Characteristics of differential expressed (DE) lncRNAs and their targets. **A**. Numbers of long noncoding RNA (lncRNA) predicted from four different platforms (databases). **B**. Ratios of different types of lncRNA. **C**. A heatmap showing expressional levels of predicted lncRNAs. **D** to **F**, Biological processes enrichment of target genes of differential expressed lncRNAs in each rice variety. **D**. ‘C34’. **E**. ‘NB’. **F**. ‘IR29’

There were 1076 expressed genes which were consistently predicted to be lncRNAs in four different prediction databases (Fig. 6A). The detected lncRNAs were composed of 61.2% lincRNA, 27.4% sense_lncRNA, 10.1% antisense_linRNA, and 1.3% interonic_lncRNA (Fig. 6B). By expressional analysis, we got 303 differential expressed lncRNAs between ‘IR29’, ‘NB’ and ‘C34’ (Fig. 6C). The differential expressed lncRNA numbers are 188, 111, and 123 in comparisons of IR29_T vs C34_T, IR29_T vs NB_T, and NB_T vs C34_T, respectively. We then employed ‘gplot’ package in R program (www.r-project.org) to generate a heatmap with clustering function (based on similarity) to measure the distance of expressional patterns of alternatively spliced transcripts around different samples, and found that lncRNA expressional patterns from same varieties with and without salt treatment grouped together. Furthermore, expressional patterns of ‘IR29’ were separated form that of ‘NB’ and ‘C34’. These results indicated that lncRNA expression variations between samples were greater than that between treatments. Moreover, more similarity in expressional pattern of lncRNA between the two salt-tolerant rice varieties.

Potential targets of lncRNA were subset into two group, *cis* targets and *trans*-targets, which were predicted through the gene locus and the sequence similarity with the master lncRNA sequence, respectively. GO analysis demonstrated that potential targets of differentially expressed lncRNAs in ‘C34’ were highly enriched in the phenylpropanoid biosynthetic process. Different from ‘C34’, candidate targets of differentially expressed lncRNAs in the other salt tolerant variety ‘NB’ were enriched in photosynthesis, light reaction and cysteine biosynthetic process. And the salt sensitive ‘IR29’ were enriched in some other biological processes, such as amino acid transport, dicarboxylic acid transport. These results suggested that salt stress disturbed lncRNA may mainly affect metabolic processes that involved in phenylpropanoid synthesis in ‘C34’ (Fig. 6D–F, Additional file 9: Table).

### Coding genes and lncRNAs Underwent AS Regulation in Different Rice Varieties

**Fig. 7:**
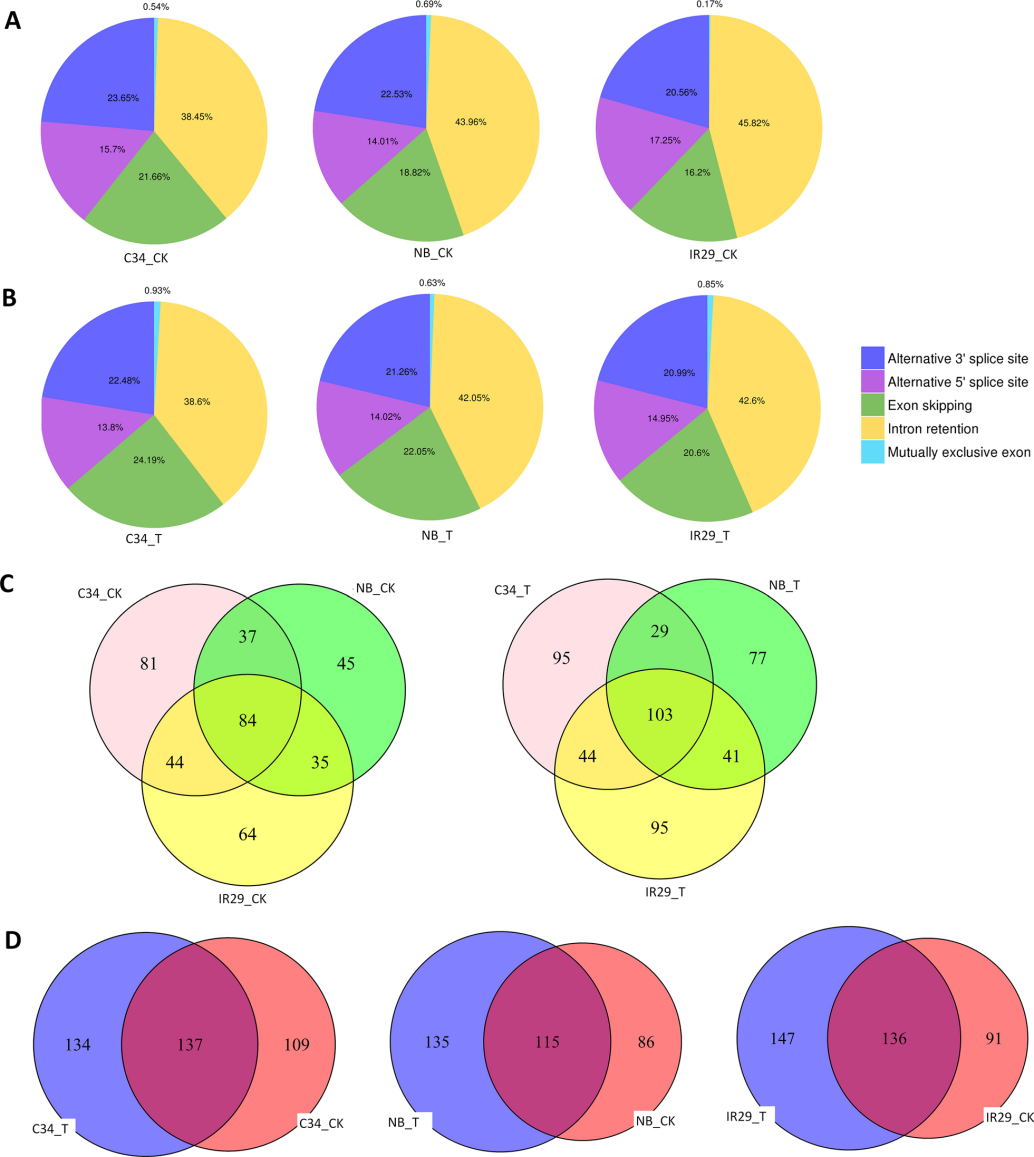
mRNA alternative splicing regulation in different rice varieties. **A**. Percentages of AS events in different rice varieties under normal conditions. **B**. Percentages of AS events in different rice varieties under salt stress conditions. **C**. Numbers of common and variety-specific Alternative spliced genes between different rice varieties under control conditions (left) and salt stress conditions (right). **D**. Numbers of alternative spliced genes in different rice varieties before and after salt treatment

In general, the AS types in plant are usually intron retentions (Ling et al. 2018; Shankar et al. 2016). Here, there were also about 40% of AS events like intron retention. Exon skipping, Alternative 3’ splice site and Alternative 5’ splice site events ranged from 13.8 to 24.2% in different rice varieties under both normal and salt stress conditions (Fig. 7 A, B). Based on annotation of sequence output with known reference mRNA isoforms, 341, 257, 287, 225, 305 and 287 AS events were detected in ‘IR29’_Salt treated, ‘IR29’_Control, ‘NB’_Salt treated, ‘NB’_Control, ‘C34’_Salt treated and ‘C34’_Control, respectively. There were a group of genes underwent AS commonly in all three rice veriaties, and also each rice variety carried out specific AS events both before and after salt stress treatment (Fig. 7C). Since most of these transcripts were expressed all rice varieties, therefore the varied abundances of different mRNA isoforms were most likely caused by variations at the mRNA splicing level (Additional file 9: Table). Furthermore, a considerable number of treatment-specific AS events were also detected in each rice variety (Fig. 7D). These results indicated that there were genotype-specific and also treatment-specific AS regulations on a considerable number of genes in rice. Notably, a considerable number of novel AS events could not be measured because of the limitation of the AS analysis used, which were carried out by using known transcripts as references.

### Validation of RNA-Seq Results Through PCR

**Fig. 8:**
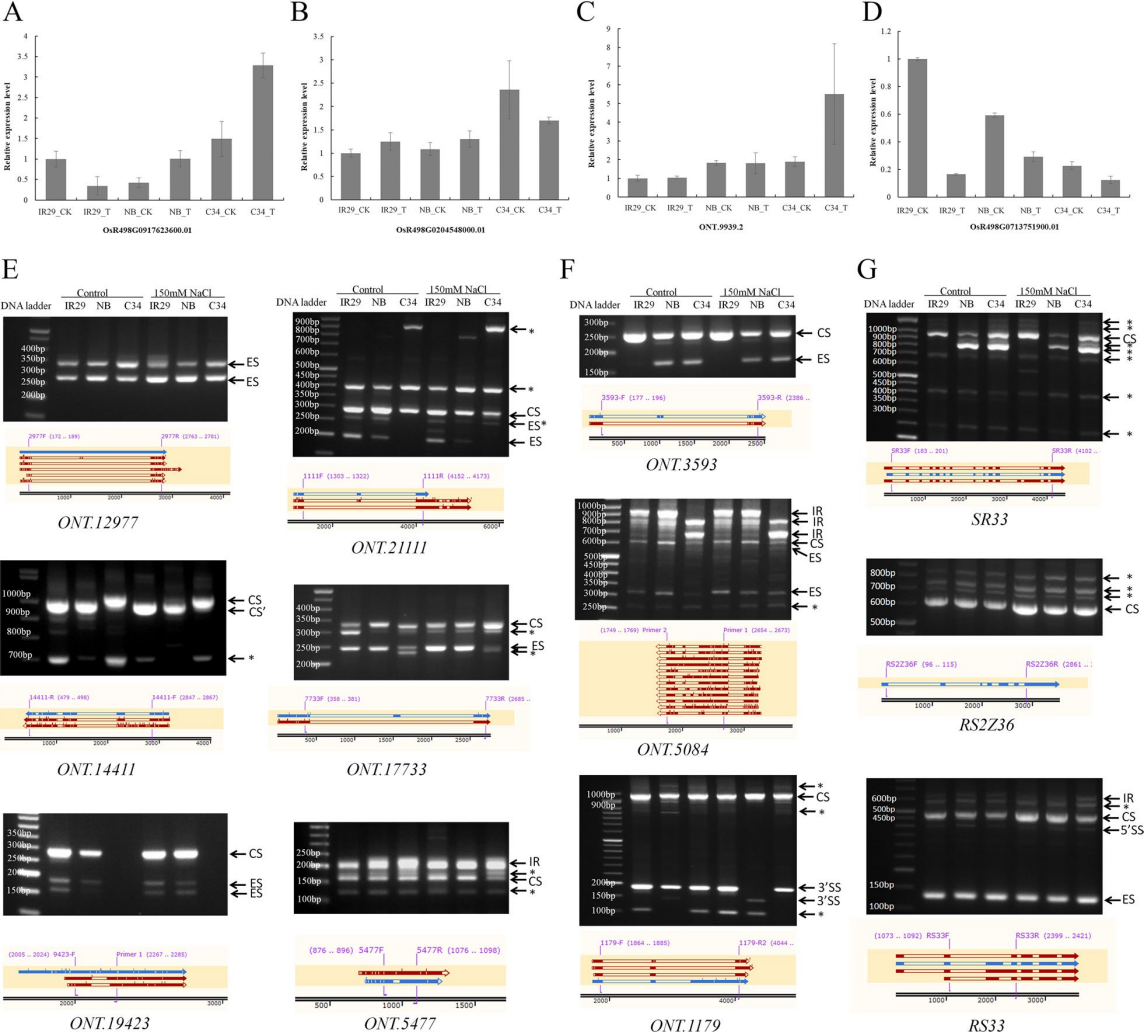
PCR validation of RNA-Seq data. Several differential expressed genes detected in RNA-Seq data were used to do qRT-PCR validation. **A** and **B**. Two salt-induced genes were expressed at higher levels in ‘C34’. **C**. A lncRNA induced significant induced in ‘C34’ by salt stress. **D**. A salt-suppressed gene show less expression in ‘C34’ when compared with ‘NB’ and ‘IR29’. **E**–**G**. RT-PCR test pre-mRNA alternative splicing events. Schematic diagram generated from comparison of genome sequence, supposed reference mRNA sequence and sequencing results in SnapGene program is shown below each RT-PCR gel electrophoresis. The AS type of PCR amplified fragment was marked by arrows with legend. CS: Constitutive splicing; ES: Exon skipping; IR: Intron retention; 3’SS: Alternative 3’ splice site; 5’SS: Alternative 5’ splice site; “*” means AS type needs further determined. **E**. RT-PCR validation of alternative splicing of coding genes. **F**. RT-PCR validation of alternative splicing of lncRNAs. **G**. RT-PCR test pre-mRNA splicing patterns of SR genes

Expression patterns of genes tested through qRT-PCR were similar to that of the RNA-Seq data in general (Fig. 8). Expression levels of a group of predicted *cis*-targets of a lncRNA, ONT.9939.2, were observed from the RNA-Seq data. Expression levels of these genes were changed after salt stress treatment in different rice varieties. However, the expression change trend of these *cis*-target genes is not strictly consistent with or opposite to the expression of their master lncRNA ONT. 9939.2 (Fig. 8, Additional file 8: Fig. S8).

Next, we validated several AS events detected on coding genes through RT-PCR. It was demonstrated that several isoforms could be amplified by using an intron-flanking primer pair of *ONT.21111.7*. All cDNA samples collected from either control or after salt treatment could amplify at least two fragments with different sizes (around 280 bp and 400 bp). Additionally, two shorter bands (around 260 bp and 190 bp) were clearly amplified from cDNA samples of ‘IR29’_Salt and ‘IR29’_Control, ‘NB’_Control. In contrast, there was a bigger transcript fragment (around 900 bp) expressed only in ‘C34’ both before and after salt treatment. However, the ‘C34’-specific transcript was highly induced by salt stress. On the other hand there was suppression of one of the common expressed transcript isoform (280 bp). AS events were also detected through RT-PCR on other genes, most of which were presented as the varied ratio between different isoforms (Fig. 8E). Similarly, variety-specific differential AS events were also detected on lncRNAs (Fig. 8E). These results indicated that both coding genes and lncRNA underwent AS regulation in rice. And interestingly, a considerable number of AS genes are present in a variety-specific manner, whereas AS variations between before- and after- salt treatment were less obvious in many genes (data not shown). Since there were few studies focused on AS regulation of coding genes and lncRNAs in response to salt stress in rice, how and to what extent such AS activities affect salt tolerance in different rice varieties needs further exploration.

A considerable number of SR genes tend to undergo AS under both control conditions and in response to salt stress, more than one target bands could be amplified through RT-PCR using intron(s)-flanking primer pair in our study (Fig. 8G). Salt treatment may induce or down-regulate the expression of specific mRNA isoform(s) of many genes, such as *RS2Z36* and *RS33*. Interestingly, *SR33* showed totally different pre-mRNA splicing patterns among the three rice varieties. ‘IR29’ expressed only a long isoform of *SR33* which was induced by salt stress. ‘NB’ expressed two isoforms under control conditions, and the shorter isoform was expressed in a higher level. However, both isoforms of this gene were down-regulated by salt stress in ‘NB’. ‘C34’ expressed the mRNA two isoforms at a similar level, and the longer isoform were down-regulated by salt stress, while the shorter one kept a relative stable expression after salt stress.

## Discussion

Generation of salt-tolerant rice with high yield and quality is still a big challenge (Qin et al. 2020). Salt tolerance is a quantitative trait that controlled by many genes, and it is suggested that most of salt tolerant rice varieties possess only a subset of salt tolerant traits (Razzaque et al. 2019) (Oomen et al. 2012). Recently, we suggested there were novel salt-tolerant rice landraces in South China (Hu et al. 2020). Uncovering the salt tolerance mechanism in different rice varieties and integrating them into an elite variety could be a solution for production of high yielding salt-tolerant rice in future.

Through transcriptome analyses, many differential regulated genes were found between rice varieties possessing varied salt tolerance in the past decade (Chao et al. 2005; Formentin et al. 2018; Li et al. 2017; Prusty et al. 2018; Walia et al. 2005). Since most of these studies compared the transcriptomes after relative long term of salt stress, gene expressional changes likely revealed the ‘outcome’ of salt damage in different rice varieties, but not illustrate how different rice respond to salt stress signals at the beginning of root environment changes (Sun et al. 2012). After 6 h of salt stress, many metabolic processes were regulated at transcriptome level in all three rice varieties. Such biological processes included carbohydrate and amino acid metabolic processes, abiotic stimuli response, photosynthesis, water shortage signaling. However, differences between the three rice varieties were mainly reflected by the intensity of regulations, including the level of up- and down-regulation of a same gene, and also the number of regulated genes from a same metabolic pathway. There are still many details to be further uncovered to fill the gap between differences in the early responses at transcriptome level of these three rice varieties and their different fates after long-term salt stress.

It has been reported repeatedly that salt stress initially causes osmotic stress. Over-accumulation of salts initially causes osmotic stress in plant roots, then ROS is generated by osmotic stress, followed by ionic imbalance and Na^+^ toxicity (Du et al. 2019; Hu et al. 2020; Li et al. 2017). Although we did not test the concentration of Na^+^ ions in the leaves within 6 h after NaCl treatment, all the three rice genotypes could sense osmotic stress by within 6 h. This is shown by the common up-regulated genes involved in the water response process (Fig. 3A). This result indicated that modulation in water uptake and allocation is one of the common and initial activities in both salt tolerant- and sensitive varieties after sensing of the salt stress signal from roots.

Excessive Na^+^ toxicity and ROS accumulation would inhibit the photosynthetic system significantly and which may cause more severe oxidative stress because of disruption of photosynthesis (Munns and Tester 2008). Thereafter, upregulation of enzymes and non-enzymatic antioxidants responding for suppressing ROS over-accumulation directly or indirectly would facilitate rice plants adapting to salt stress (Kaur et al. 2016; Steffens 2014; Wang et al. 2018b; Zhou et al. 2018). In an earlier study, overexpression of ASCORBATE PEROXIDASE (APX) genes enhanced salt tolerance in rice (Hong et al. 2007). Furthermore, suppression of genes involved in ROS biosynthesis was also suggested to be a potential method for improving salt tolerance in rice (Qin et al. 2020). Here, the ROS contents of two salt tolerant rice varieties ‘C34’ and ‘NB’ were significantly lower than that of ‘IR29’ after 5 days of salt stress. Interestingly, one of AXP genes in rice, *AXP1*, was induced to higher levels by salt treatment for 6 h in ‘C34’in ‘NB’. Moreover, some other genes involved in ROS scavenging were specifically induced in ‘C34’. These findings suggested that stronger induction of anti-oxidant genes could be one of salt tolerant characteristics of ‘C34’.

It is a known conclusion which has been described in many publications that salt stress inhibited photosynthetic process in plants (do Amaral et al. 2016; Munns and Tester 2008). ‘C34’ and ‘NB’ leaves possess higher content of chloroplast in continuous salt stress conditions for several days when compared with ‘IR29’ (Hu et al. 2020). Previous studies also demonstrated that relative high level of photosynthesis activity correlates to higher levels of salt tolerance. And at transcriptional level, most of these studies measured genes expression patterns at relative long-term after salt stress and detected higher expression levels of genes involving in photosynthesis in the tolerant genotypes (Formentin et al. 2018; Li et al. 2017; Prusty et al. 2018; Walia et al. 2005). In our study, we found that more down-regulation of genes involving in photosynthesis and chloroplast in the two salt tolerant rice varieties, ‘C34’ and ‘NB’, when compared with ‘IR29’. We assume that the expression of photosynthesis genes may be regulated differentially when the salt stress prolonged, and different rice varieties may respond at different rates. This group of genes may be down-regulated faster in the salt tolerant genotypes than in the sensitive ones, and their expressions can be recovered to relative higher levels when they adapt to the salt stress. In salt sensitive genotypes, such as IR28 and IR29, down-regulation of these genes was carried out slower, and if the seedlings can’t recover from salt stress, expression of genes will not recover any more. To confirm this assumption, comprehensive comparisons of gene expression changes in time course should be done between contrasting salt tolerance genotypes. However, the phenomenon of slower response in some biological processes in IR29 when suffering salt stress supported, to a certain extent, the possibility of our assumption (Moradi and Ismail 2007). Supportively, more intensive down-regulation of genes involving in photosynthesis and chloroplast in salt tolerant genotypes was also found in other species. When compared gene expressional patterns in different Arabidopsis ecotypes, it has been found that abundance of photosynthesis genes were less in the most tolerant ecotypes Sha under salt stress, when compared with Ler and Col (Wang et al. 2013). Similarly conclusion has also been found in water melon, the salt-tolerant genotypes ‘BXC’ exhibited less reduction in transpiration rate, net photosynthesis rate, and stomatal conductance than ‘YL’(salt sensitive) under 150-mM NaCl stress. But more downregulated genes involved in photosynthesis were found in ‘BXC’ (Wang et al. 2016a). Moreover, after studying salt stress response in different barley varieties, it is proposed that a reduction in photosynthesis and respiration resulting in utilization of the stored energy and the carbon for maintaining plant organs, could be a mechanism of salt tolerance in plants (Yousefirad et al. 2020).

These findings suggest that turning down photosynthesis in rice at early stage of salt stress could be a protective mechanism. Moreover, such mechanism may be more prevalent in tolerant varieties. Such positive modulation will better protect leaves from oxidative stress in rice if the salt stress is prolonged by shutting down photosynthetic activity and ROS generation through the photosynthetic system. Consequently, reduction of ROS will mitigate the salt stress-induced damage of cellular membrane systems. How this protective shutdown of photosynthetic system occurs is not very clear. Probably there could be a possibility that the down-regulation of photosynthesis is controlled by the ROS burst generated immediately after the salt-induced osmotic stress in the root. A recent study found that a quick accumulation of ROS in the root at early stage of salt stress could be part of a coordinated response to facilitate salt adaptation, but not the reason of salt-induced leaf senescence (Formentin et al. 2018). Supportively, it has been concluded in a recent review that reduction in photosynthesis activity is a regulatory mechanism when plants suffering stress conditions (Zhang et al. 2020). Therefore, our study suggests that salt-tolerant rice varieties may evolve a protective mechanism to turn-down the photosynthesis activity to a basic level for preventing over-accumulation of ROS under salt stress.

Retardation of growth and productivity in various plants were partially caused by stress inhibition of ribosomal protein (Moin et al. 2017; Mukhopadhyay et al. 2011, 2013). In our study, genes encoding ribosomal proteins were down-regulated at 6 h after salt stress, and the number of regulated genes in ‘IR29’ was greater than that of ‘C34’ and ‘NB’ (Fig. 2D, E). We supposed that leaves of these salt-treated plants sensed the stress, and then the down-regulation of ribosomal proteins may cause protein biosynthesis inhibition. This inhibition was carried out more obviously in ‘IR29’, which could be a reason why ‘IR29’ leaves show more severe senescent phenotype when the salt stress prolonged (Hu et al. 2020). Supportively, it has been found that overexpression of some ribosomal proteins, including a ribosome inactivating protein, OsRIP18 (Jiang et al. 2012) and a ribosomal protein RPL6 (Moin et al. 2021), enhanced salt and drought tolerances of the transgenic rice. Moreover, ACC deaminase producing bacteria-inoculated plants showed enhancing stress tolerance, which could be attributed to higher abundance of ribosomal proteins after the inoculation (Roy Choudhury et al. 2022). So, less inhibition of genes involved in ribosome in ‘C34’ and ‘NB’ would be one of reasons of their enhanced salt tolerance.

Important roles of Na^+^ and K^+^ transporters for salt tolerance in rice has been discussed in many studies (Xie et al. 2022). Increased activity of Na^+^ transporters through either amino acid sequence changes or protein expression change that enduring relative low accumulation of Na^+^ in the cytoplasm of cells could lighten the Na^+^ toxicity and finally increase the salt tolerance of plants (Kobayashi et al. 2017; Obata et al. 2007; Oomen et al. 2012; Ren et al. 2005; Shen et al. 2015). In a study on rice, two potassium transporters *LOC_Os02g49760.1*and *LOC_Os04g51190*, were specifically up-regulated under salt stress in ‘FL478’ (a rice variety possessing salt tolerant features of ‘Pokkali’), but not in its salt sensitive parent ‘IR29’. Therefore these two genes were supposed to be salt-tolerant contributor of FL478 (Mirdar Mansuri et al. 2019). However, expressional patterns of these two genes didn’t show obvious differences among the rice varieties in our study. *LOC_Os02g49760.1* was induced in all three rice varieties after salt stress, whereas *LOC_Os04g51190* was expressed constitutively (Fig. 5). The candidate gene for salt tolerance QTL in rice variety ‘NB’ was considered to be *SKC1*, whose target gene encodes a member of High-Affinity Potassium Transporters (HKTs) (Oomen et al. 2012; Ren et al. 2005). Other studies have also detected the important roles of HKTs (Hartley et al. 2020; Hauser and Horie 2010; Horie et al. 2007; Huang et al. 2008; Suzuki et al. 2016; Yao et al. 2010). In our experiment, one of HKT genes, *OsHKT1;1,* was induced by salt treatment in all three rice varieties, with the highest induction in ‘NB’, followed by ‘C34’. However, a low-affinity Na^+^ transporter *OsHKT1;3* (Rosas-Santiago et al. 2015) was down-regulated most significantly in C34, when compared with ‘NB’ and ‘IR29’ (Fig. 5). These results indicated that expression of some Na^+^/K^+^ transporters in leaves of 3 rice genotypes were regulated differentially at early stage of salt stress.

A salt tolerant rice cultivar ‘Changmaogu’ showed stronger development in shoot under salt stress at germination stage. Sun et al. found there were 169 overlapping DEGs in rice root among three time point (30 min, 3 h and 24 h) of salt treatments, 13 and 6 of these DEGs were involved in ABA signaling and carotenoid biosynthesis. One of DEGs *OsPP2C8* (Os1g0656200) showed sequence polymorphism in rice with different levels of salt tolerance. And *OsPP2C08* (Os01g0656200) was identified as the candidate gene for salt tolerance in ‘Changmaogu’ (Sun et al. 2019). In our study, another PP2C gene, *OsPP2C27*, was induced to higher levels in leaves of the two salt tolerant rice varieties, especially in ‘C34’, suggesting ABA signaling were carried out differentially in leaves at this point time (6 h after salt stress). Different ABA signaling genes may be used for salt stress response in different organs of rice. Consistently, there was a group of stress-responsive transcription factors differentially expressed at this stage of salt stress in the three rice varieties in our study (Fig. 4A).

lncRNAs were considered to be important components in the stress response in rice. A long noncoding RNA, LDMAR, regulates photoperiod-sensitive male sterility in rice (Ding et al. 2012). In addition, there were more than 200 lncRNAs involved in drought memory response in rice (Li et al. 2019). Here, different groups of lncRNA may be induced in different rice varieties (Fig. 6C). Based on RNA-Seq analysis, the differentially expressed lncRNAs in ‘C34’ was observed to target genes involved in the phenylpropanoid biosynthesis pathway (Fig. 6D). Interestingly, several recent studies suggested regulation in metabolism of cell wall components, such as phenylpropanoid and lignin, are related to salt stress responses in different kinds of crops (Ho et al. 2020; Oliveira et al. 2020; Zhu et al. 2021). This implies that the difference in modulating phenylpropanoid synthesis could also contribute to variation in salt tolerance in the tested rice varieties. However, there were no regular patterns of variation in the expression of target candidates according to their disturbed master lncRNA (Additional file 8: Fig. S8). There could be a possibility that some candidate target genes of the master lncRNA may also be targeted by other lncRNAs, at the same time or other regulators, such as small RNAs (not measured in this study), like it has been reported on other lncRNAs (Hong et al. 2022; Li et al. 2022). Alternatively, lncRNAs may regulate their targets through other layers, including translation and protein modifications (Fatica and Bozzoni 2014; Fonouni-Farde et al. 2021). Moreover, the post-transcriptional regulation of the master lncRNA itself may also control its binding activity (Yuan et al. 2018), so induction of different isoforms of master lncRNA may change its preference of targeting. Uncovering how lncRNA-targets regulatory networks function under salt stress condition is challenge but import for understanding the mechanism underlying salt stress response in rice.

Accumulation g of evidence proved that AS regulation was essential for stress tolerance and adaptation in plants. In Arabidopsis, AS and mRNA maturation of salt-tolerance genes were disturbed in splicing-related mutants (Feng et al. 2015). Furthermore, mutation in a U1 spliceosome protein, AtU1A, affect the splicing process of many genes, including the ROS detoxification-related gene ACO1, which is considered to cause reactive oxygen species (ROS) over-accumulation and be hypersensitive to salt stress in the *atu1a* mutant (Gu et al. 2018). In Rice, gene transcriptional and AS regulation after salt and drought stress were studied years ago (Shankar et al. 2016). Interestingly, they demonstrated that the frequency of different AS events under control and stress conditions within each rice cultivar were similar to the AS events predicted at the whole transcriptome level. Furthermore, the frequency of AS events was less under stress conditions. A group of superoxide dismutases (SODs) gene underwent AS regulation in a tissue-specific and stress-specific manner, which were supposed to be important for rice adaptation to stresses (Saini et al. 2021). Recently, through data mining from hundreds of RNA-Seq data, Yu et al. (2021) predicted that genotype-specific AS regulations of *OsNUC1* and *OsRAD32* were associated with different responses of upper part to salt stress in rice population. Here, we found groups of genes underwent AS in rice (Figs. 7A–D and 8E–G). Intron retention (IR) is the most popular AS event like it has been found in other studies (Fig. 7A). Interestingly, there were genotype-specific AS in rice, both before and after salt stress. For example, different isoforms of *ONT.12977.2*, *ONT.21111.7* and *ONT.14411.5* were expressed in the three rice varieties (Fig. 8E). Not only encoding genes, but also lncRNAs could carry out variety-specific AS in rice. And IR was also the most popular AS event on lncRNAs Fig. 8F). However, we do not know the functional role of AS regulation of lncRNA until now. Does the inclusion of intron(s) affect target-binding or the stability of the lncRNA itself? It is a question need to be answered to totally the mechanism of AS regulation on noncoding RNAs. Nevertheless, the phenomenon we found here implied that the pre-mRNA splicing regulatory modulation could regulate protein expression directly and indirectly through multiple pathways in rice.

The active expressional change and AS regulation was carried out on encoding genes of splicing factors, including that of SR proteins, in response to kinds of stresses. Such AS regulation were supposed to amplify the effect of AS regulation on genome wide in the suffering cells through the molecular function of SR proteins (Palusa et al. 2007). Most recently, it has been demonstrated that mutation of a SR protein in rice disturbed pre-mRNA splicing of some genes and affect cold and salt tolerances of rice (Butt et al. 2022). As we found that there are variety-specific AS regulation were carried out on a considerate number of genes in different rice varieties, therefore variety-specific expression and/or AS of splicing factors may also participate in such regulation. By RT-PCR, we found that SR proteins underwent AS both before and after salt stress in all three rice varieties (Fig. 8G). The smallest isoform of *RS2Z36* was significantly induced by salt stress in all varieties. More interestingly, a splicing factor *SR33* underwent totally different AS regulations between the three different rice varieties. A fragment (about 950 bp) was significantly induced in ‘IR29’, whereas another fragment (800 bp) was highly expressed in ‘C34’ and ‘NB’, and both isoforms were suppressed by salt stress. Variety-specific regulation of mRNA splicing factors in rice to a certain extend explained why we observed the different splicing patterns on the same genes, including coding and noncoding, in the three rice varieties. Variety-specific AS regulation of mRNA, together with Variety-specific transcription, could be finally contributed to different response to salt stress in different rice varieties.

**Fig. 9:**
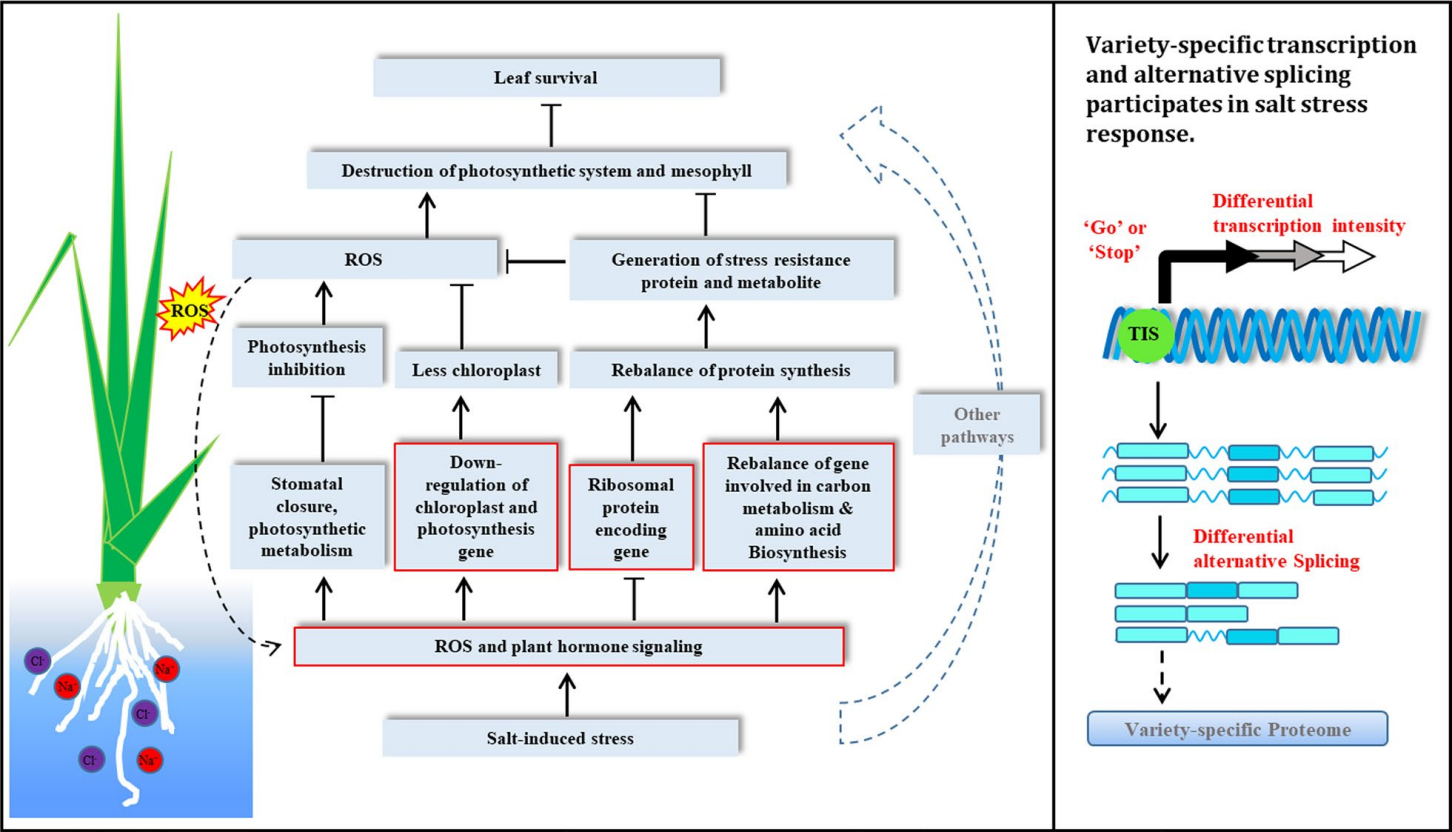
A landscape of molecular response in rice leaves at early stage of salt stress. The left panel: The salt treatment induced water shortage, ABA signaling and ROS burst. These signals would affect upper part of rice plants through several different pathways after several hours of salt stress. (1) ABA and ROS transports to leaves and close stomata and disturb photosynthetic metabolism, which will cause over excessive generation of ROS, and subsequently destroy the membrane system of cells if the ROS cleavage system does not function efficiency, finally make cell death and leave senescence (Apel and Hirt 2004; Chaves et al. 2009; Hu et al. 2020). (2) At early stage of salt stress, water shortage triggered ROS burst down regulated chloroplast development and photosynthetic components (Formentin et al. 2018), which will subsequently reduce photosynthesis activity in leaf cells, and then reduce the ROS accumulation (Huang et al. 2009; Ponce et al. 2021; Wang et al. 2021). (3) ROS and ABA and other plant hormone signaling turn down the ribosome, which is responsible for protein synthesis. Reduction protein synthesis will reduce energy consumption of the plants (Moin et al. 2021). However, basic protein synthetic activity is still necessary, because stress proteins are required for stress tolerance. (4) Genes involved in carbohydrate and amino acids metabolism were regulated to a new balance status after sensing the ROS, ABA and other hormone signals (Ponce et al. 2021). This pathway and (3) participates in regulation of protein biosynthesis, modulating abundance of stress proteins, such as ROS cleavages (Ponce et al. 2021). The integrated regulations in different pathways at early stage of salt stress may finally control longevity of leaves under continuous salt stress conditions (Hu et al. 2020). Other regulations not discussed here could also found from (Ponce et al. 2021). The right panel: Expressions of genes responsible for these modulations could be regulated through a variety-specific manner on both transcriptional and mRNA splicing levels

In summary, our study provided a landscape of gene expression in rice leaves at early stage of salt stress. Differential regulations of genes related to chloroplast synthesis, photosynthesis, ROS generation and cleavage, and so on, these would consequently contribute to different performances of different rice varieties in the protection of chlorosis and cell damage on leaves. Such gene expressional regulations were carried out not only on coding genes, but also on noncoding genes, through both transcription and mRNA splicing levels. Based on results in this study and others (Apel and Hirt 2004; Chaves et al. 2009; Formentin et al. 2018; Hu et al. 2020; Huang et al. 2009; Moin et al. 2021; Ponce et al. 2021; Wang et al. 2021), we drew a picture to illuminate gene expressional regulations at early stage of salt stress in rice leaves and their affection on the performance of salt tolerance if the salt stress prolonged (Fig. 9).

## Supplementary Information

**Additional file 1:** Plots of RNA read length and Average reads quality.

**Additional file 2:** Plots of number of expressed transcripts and reads number (sequencing depth).

**Additional file 3:** Sample correlations between replicates and treatments in 'C34’.

**Additional file 4:** Read Counts vs. Reads Length Barplot of all samples.

**Additional file 5:** Numbers of expressed genes in three rice varieties.

**Additional file 6:** GO analysis of expressed genes in rice.

**Additional file 7:** Biological process enrichment of common and variety-specific regulated genes.

**Additional file 8:** Expression patterns of genes within 200 Kb around the predicted IncRNA ONT.9939.2.

**Additional file 9:** Supplementary tables for more detailed information for RNA-Seq data analyses.

## Data Availability

All data generated or analyzed during this study are included in this published article and its supplementary information files.
