# Supplementary figures and images for: Variety-Specific Transcriptional and Alternative Splicing Regulations Modulate Salt Tolerance in Rice from Early Stage of Stress
Source: Rice (N Y). 2022 Nov 3;15:56. doi: 10.1186/s12284-022-00599-9 (PMC9633917; doi:10.1186/s12284-022-00599-9)

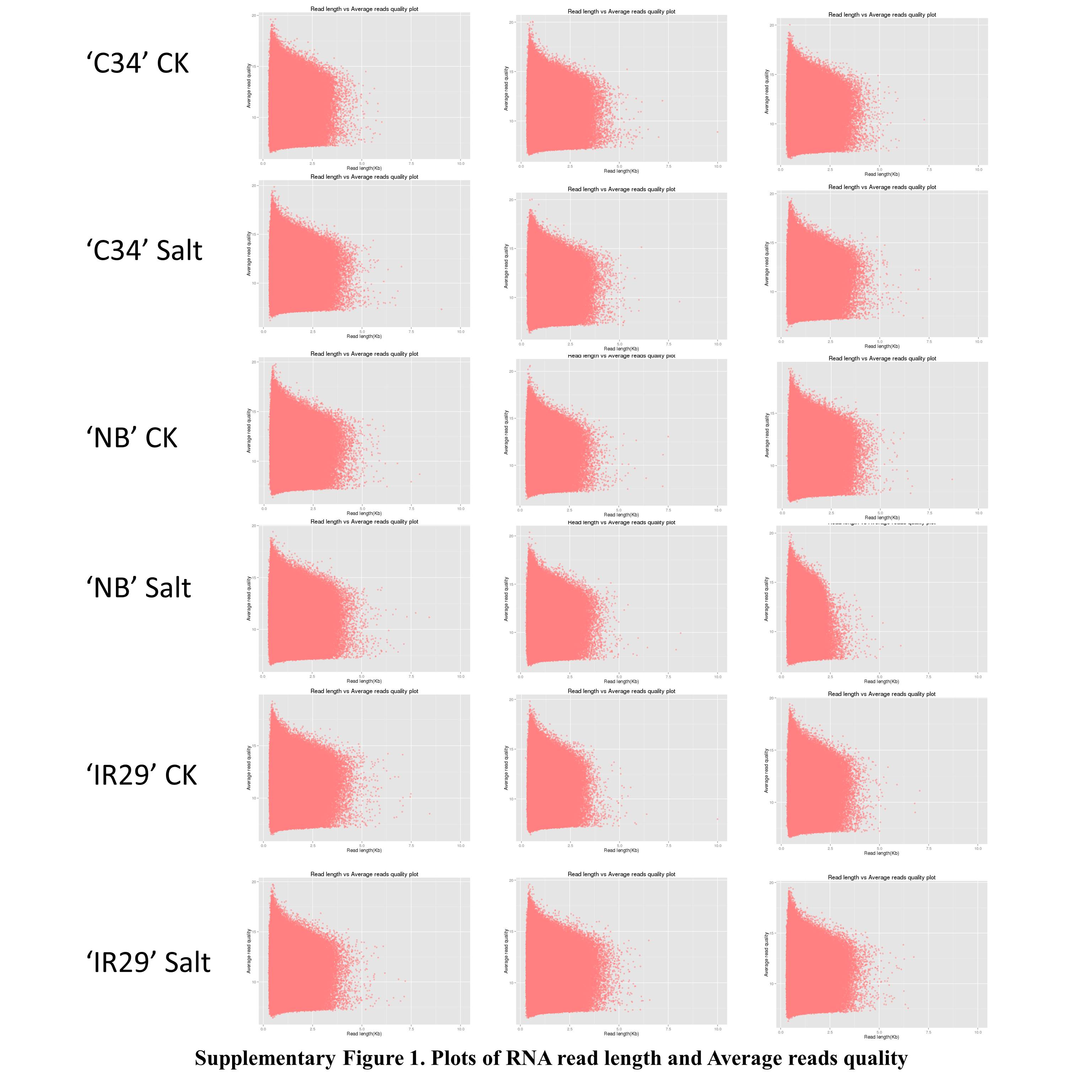

Supplement: Supplementary file 1 — Additional file 1: Plots of RNA read length and Average reads quality. [file 12284_2022_599_MOESM1_ESM.jpg]

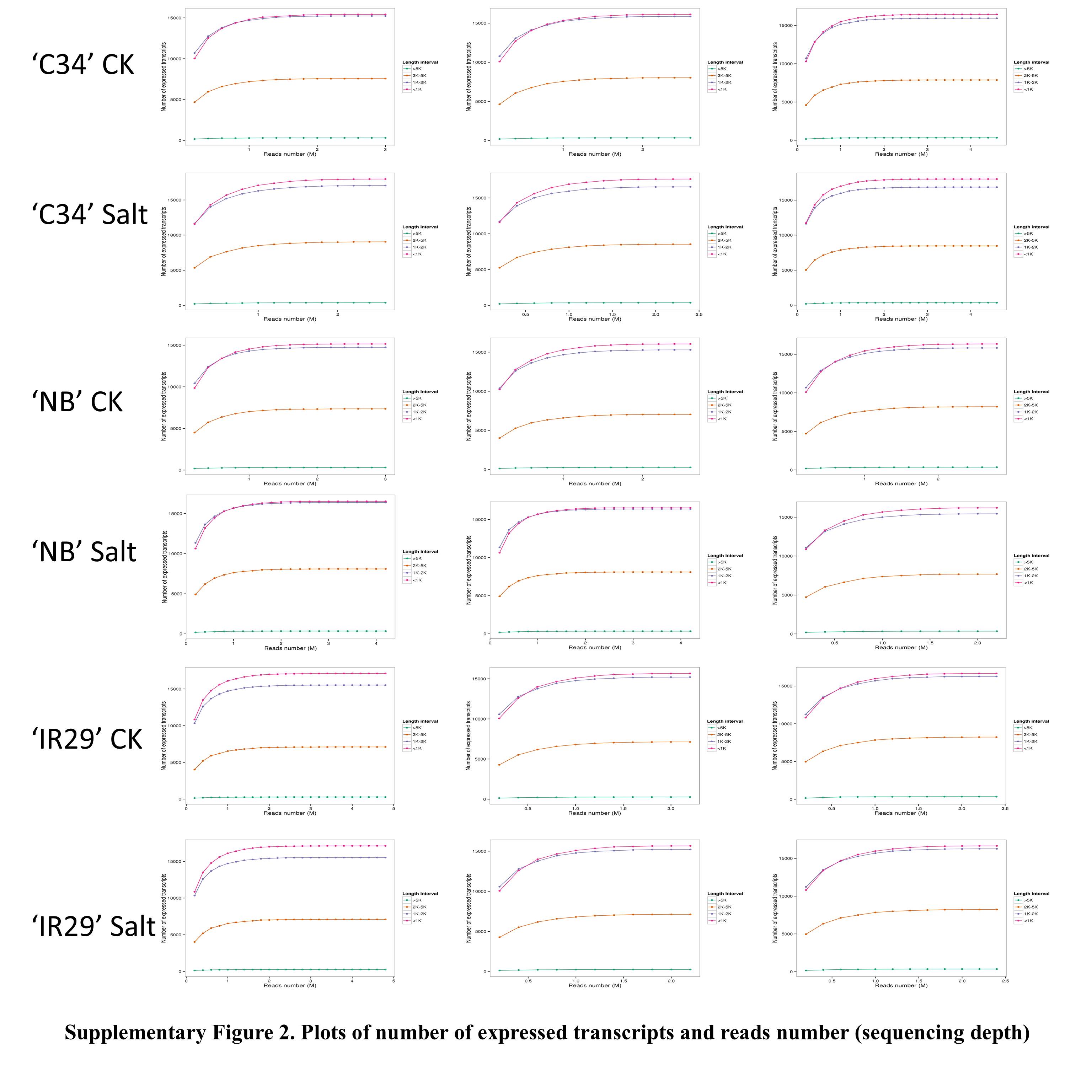

Supplement: Supplementary file 2 — Additional file 2: Plots of number of expressed transcripts and reads number (sequencing depth). [file 12284_2022_599_MOESM2_ESM.jpg]

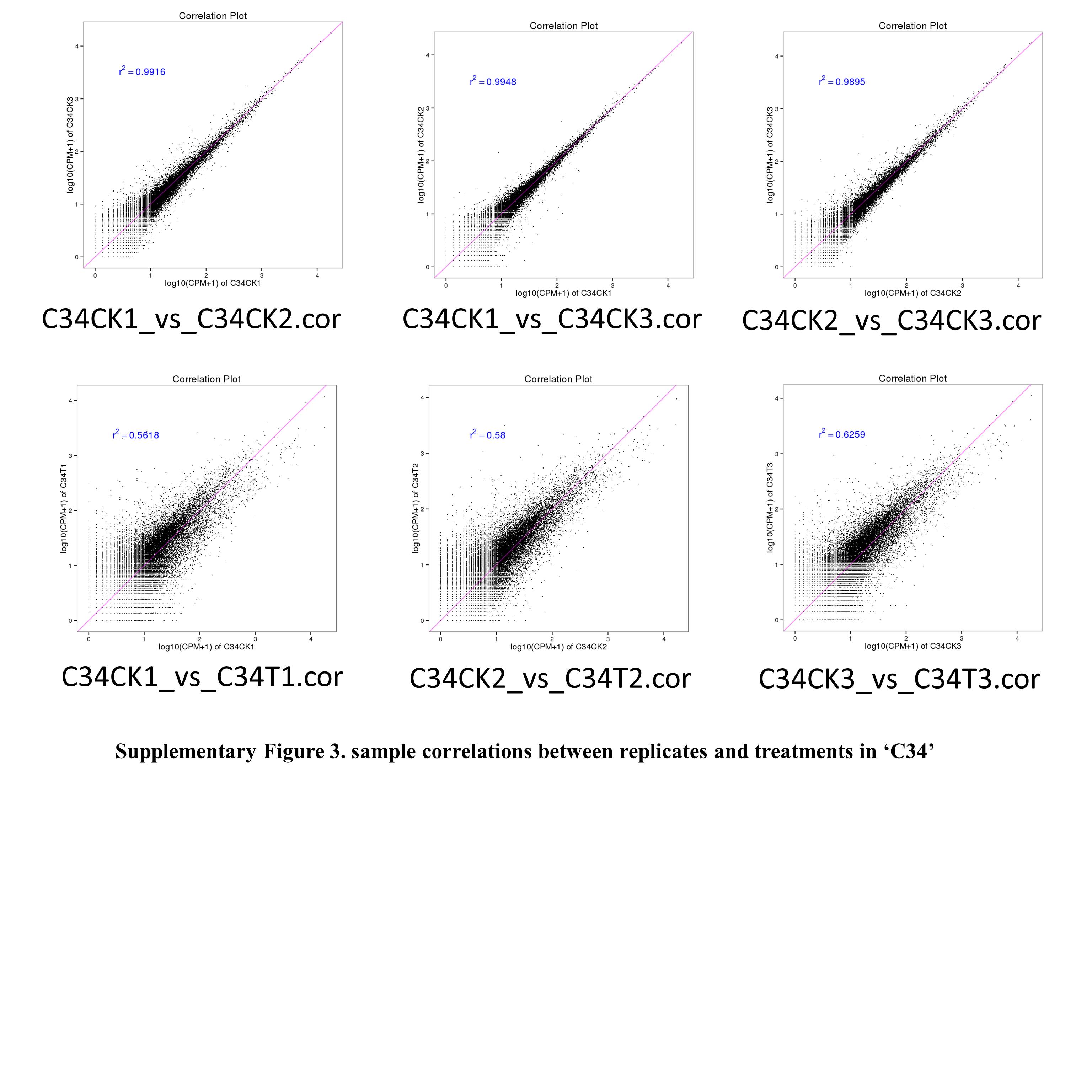

Supplement: Supplementary file 3 — Additional file 3: Sample correlations between replicates and treatments in 'C34’. [file 12284_2022_599_MOESM3_ESM.jpg]

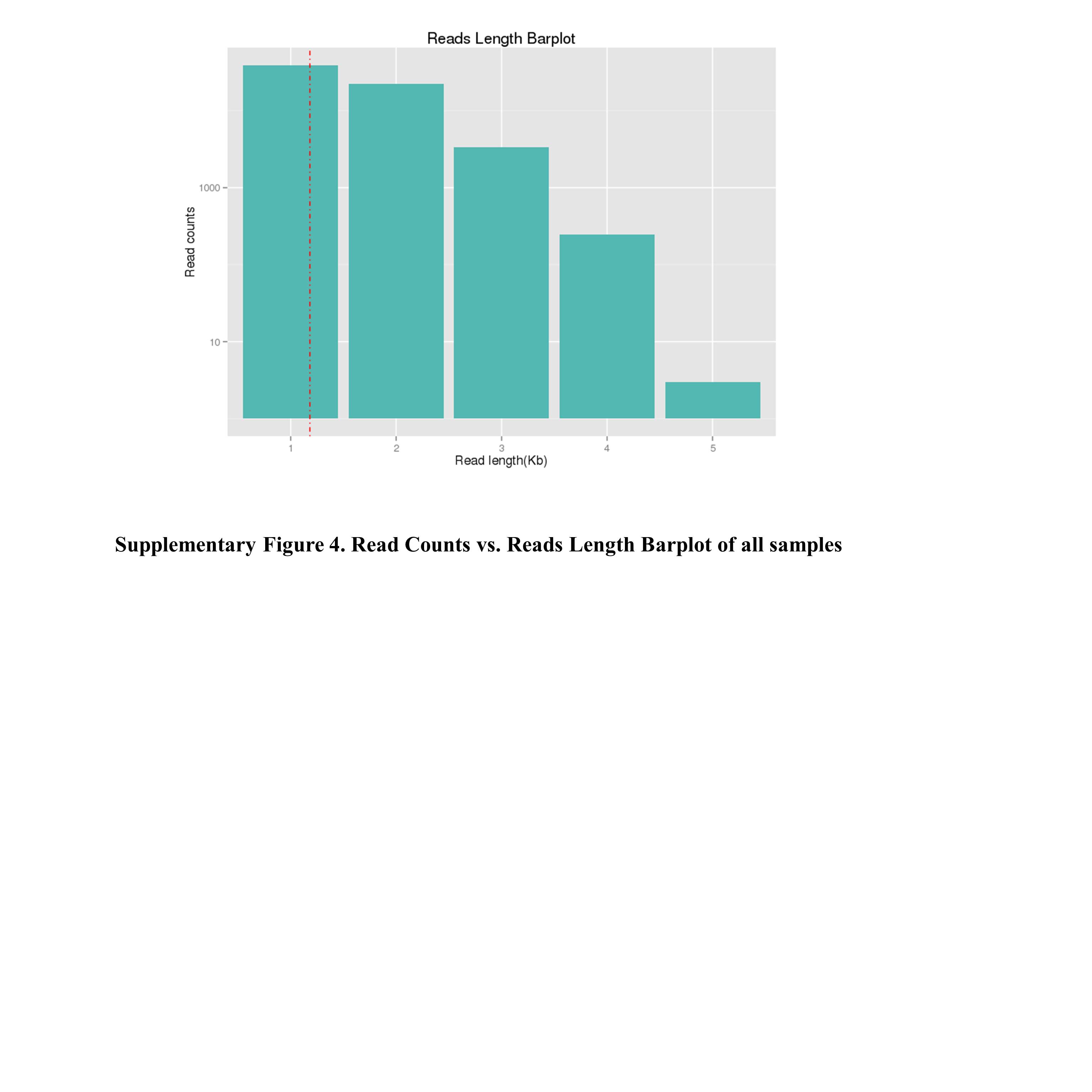

Supplement: Supplementary file 4 — Additional file 4: Read Counts vs. Reads Length Barplot of all samples. [file 12284_2022_599_MOESM4_ESM.jpg]

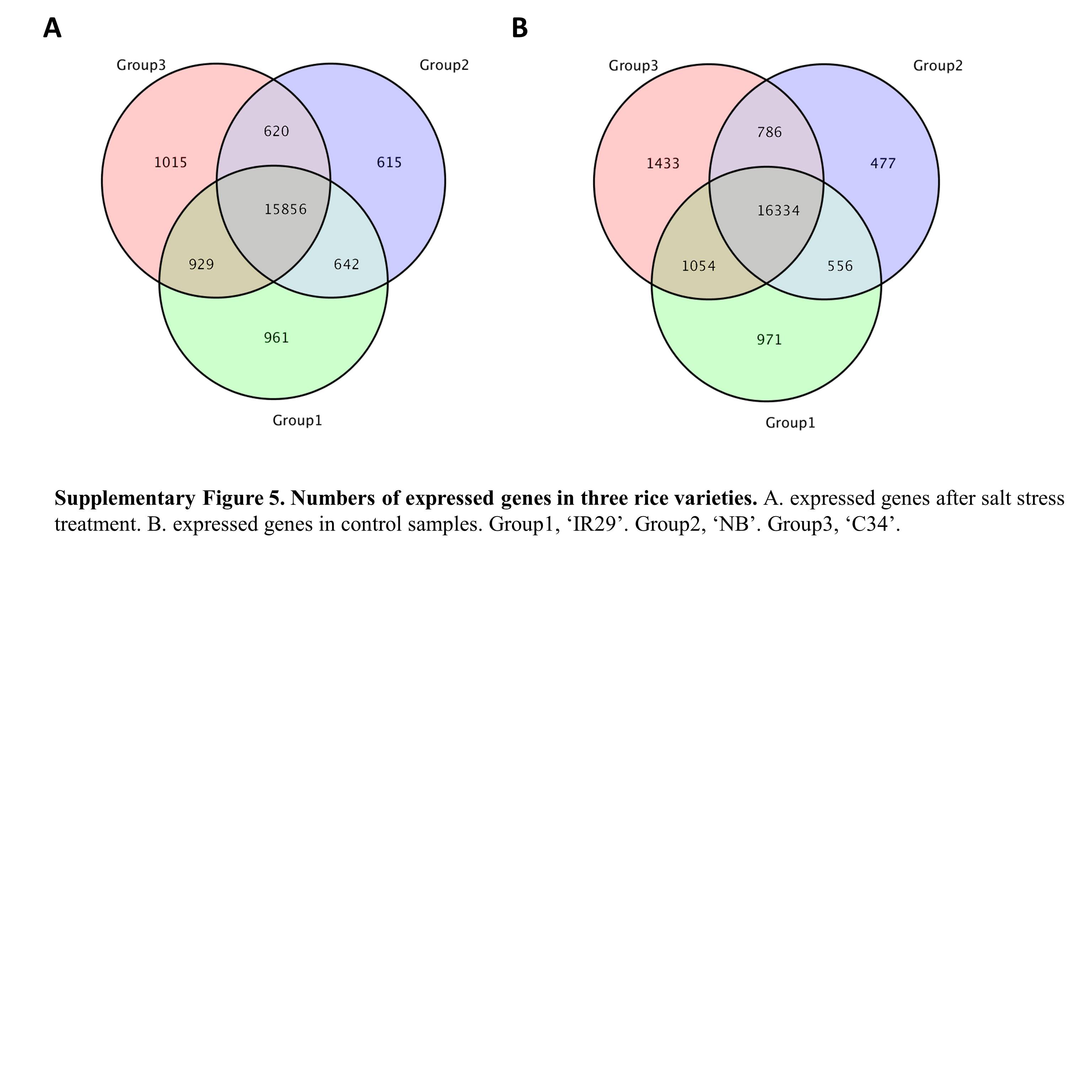

Supplement: Supplementary file 5 — Additional file 5: Numbers of expressed genes in three rice varieties. [file 12284_2022_599_MOESM5_ESM.jpg]

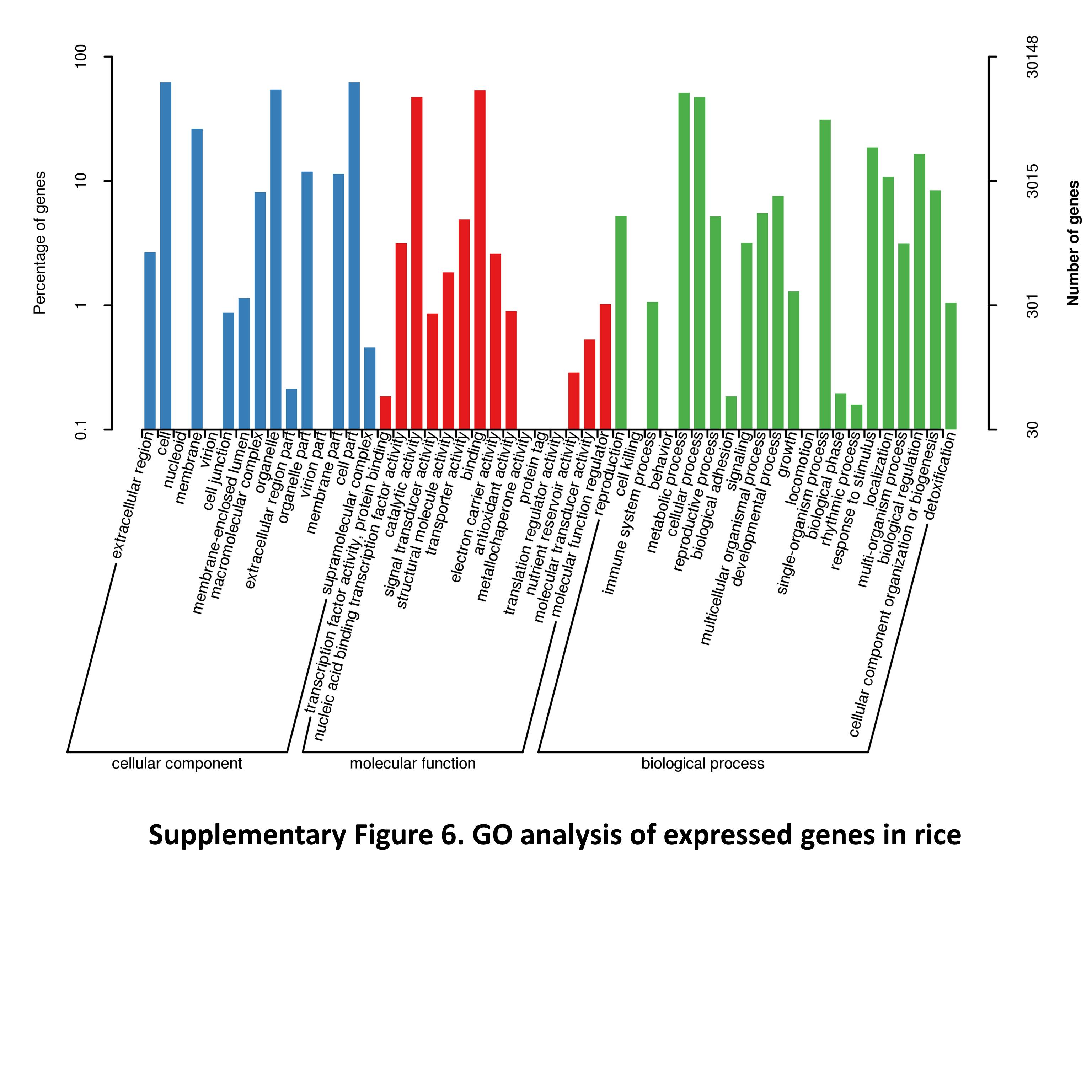

Supplement: Supplementary file 6 — Additional file 6: GO analysis of expressed genes in rice. [file 12284_2022_599_MOESM6_ESM.jpg]

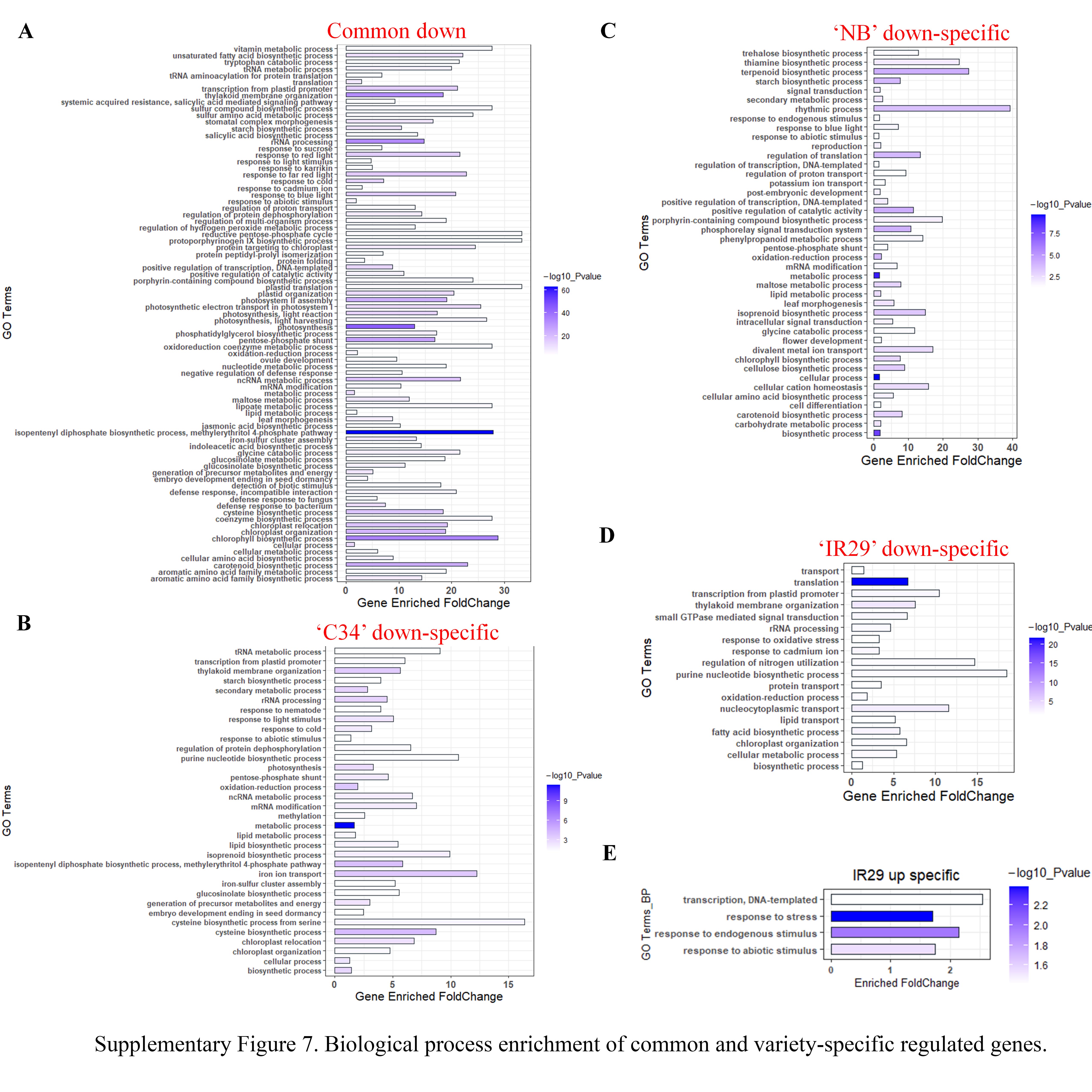

Supplement: Supplementary file 7 — Additional file 7: Biological process enrichment of common and variety-specific regulated genes. [file 12284_2022_599_MOESM7_ESM.jpg]

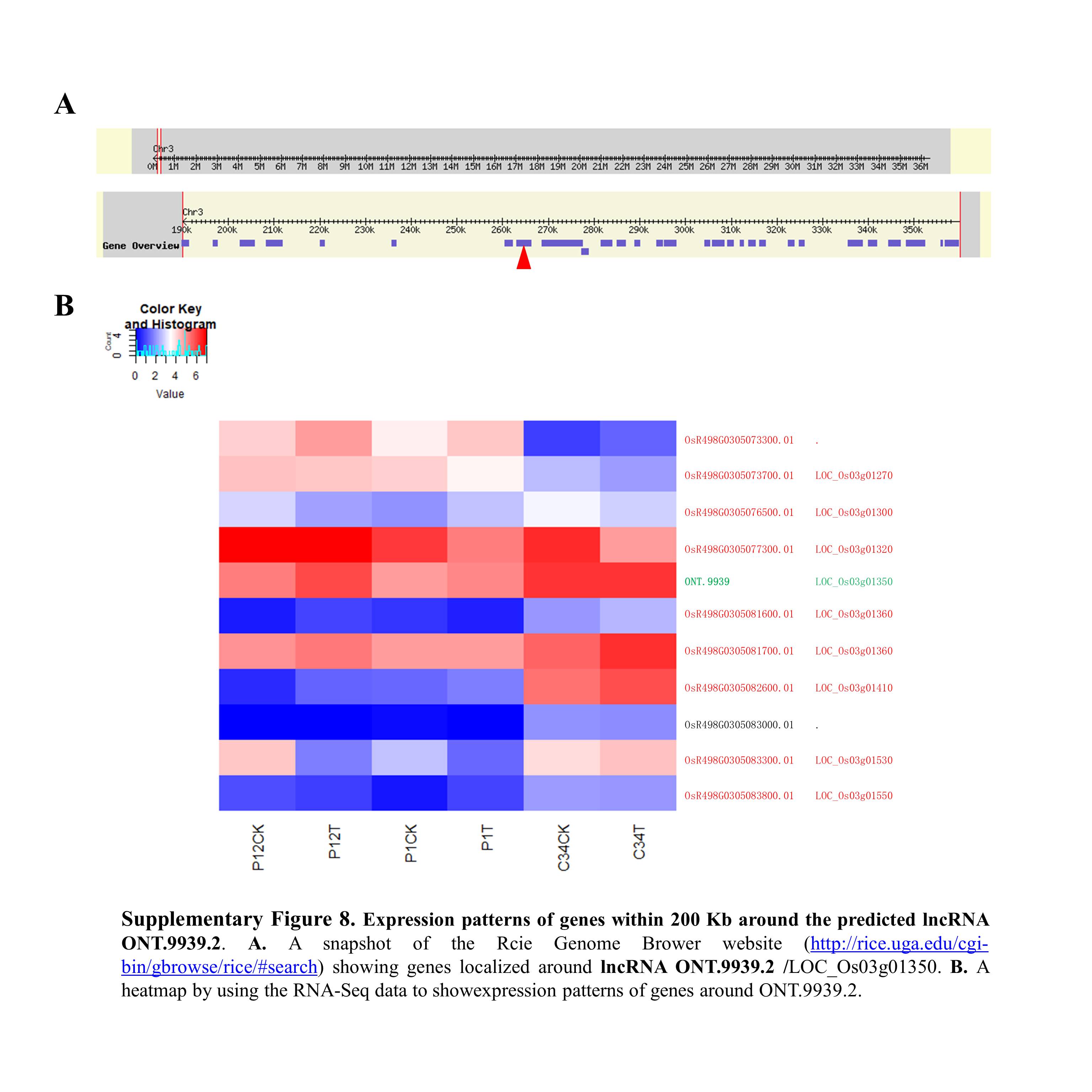

Supplement: Supplementary file 8 — Additional file 8: Expression patterns of genes within 200 Kb around the predicted IncRNA ONT.9939.2. [file 12284_2022_599_MOESM8_ESM.jpg]
